# Does the Relative Age Effect Influence Short-Term Performance and Sport Career in Team Sports? A Qualitative Systematic Review

**DOI:** 10.3389/fpsyg.2020.01947

**Published:** 2020-09-23

**Authors:** Alfonso de la Rubia, Jorge Lorenzo-Calvo, Alberto Lorenzo

**Affiliations:** Departamento de Deportes de la Facultad de Ciencias de la Actividad Física y del Deporte-INEF de la Universidad Politécnica de Madrid, Madrid, Spain

**Keywords:** relative age effect, birthdate, performance, competition, sport talent, statistics, sport success, team sport

## Abstract

**Background:** The impact on athletes based on grouping methods according to the date of birth within the constituent year, known as the relative effect of age (RAE), is a factor that can influence the achievement of sports success. Many studies have examined the magnitude of this phenomenon in sport; however, the relationship between the RAE and performance in team sports competition has not been accurately evaluated so far. The purpose of this study was to conduct a systematic review on the influence of the RAE on competition performance in team sports through analysis of published peer-reviewed articles from 2000 to 2019.

**Methods:** According to Preferred Reporting Items for Systematic Reviews and Meta-Analysis systematic search guidelines, 19 studies were identified of the 2,093 that were found in the systematic searching process carried out in four databases: Sport Discus, PubMed, Web of Knowledge, and Scopus. The sample of the study was composed by 77,329 players, of which 92.08% were male and 7.92% were female, whereas the recorded performance measurements were 87,556. The relation between relative age effects and competition performance was registered according to constraints-based theoretical model: individual constraints (sample characteristics) and task constraints (sport context). Moreover, study quality analysis, Strengthening the Reporting of Observational Studies in Epidemiology, was carried out.

**Results:** The short-term individual (10.20%) and collective (18.09%) performance was influenced by the RAE, whereas the long-term individual performance (49.71%) was affected by the RAE reverse. However, in 16.99% of the measurements, no relationship was found between the RAE and competition performance. In the analysis by subcategory, the influence of the RAE was higher in men, in adulthood (senior category), in invasion games, and in national contexts.

**Discussion:** The findings clearly demonstrated that the RAE has a great influence on the performance in team sport. Possible implications for policy and practice should be discussed in order to prevent unequal practice based on biased models that prioritize the athlete's current performance and therefore obviate their maturational development. The heterogeneity and variability of the identified results require a relativization of the findings of this study.

## Introduction

In sport, especially at the highest competitive level, there has always been a constant search to achieve excellence at the individual level. In a team sports context, this fact can also extend to the collective field (Vaeyens et al., 2008). However, one of the issues of sports talent identification programs is the attempt to recognize, through transversal measurement models, a future “talent” according to the athlete's current performance characteristics (physiological, physical, and/or anthropometric) and the current characteristics of their own sport and its evolution (Bailey and Collins, 2013). This approach seems not to take into consideration the maturational status of the athlete, which is omitted when analyzing the player's development process, or other factors connected to the effects produced by training (Abbott and Collins, 2002). Therefore, this situation could produce an imbalance, in terms of sport development, between the athlete's maturity level and his/her chronological age (Torres-Unda et al., 2013).

The grouping of athletes by age group is very common in sport. In particular, the categories in team sports usually correspond to annual or biannual competitive cycles, getting into competition groups according to the athlete's chronological age and according to a previously established cutoff date (January 1 is globally accepted as the beginning of the selection year). This normally applied strategy sharpens the differences between athletes because of their maturation status, which does not necessarily correspond to their chronological age (Wattie et al., 2008). This phenomenon is known by the name of “relative age,” and the consequences are referred to as the relative age effect (RAE) (Musch and Grondin, 2001).

This situation is usually reflected in youth sports contexts and is understood as an overrepresentation of athletes born in the first months of the year because of a greater maturational development (Barnsley et al., 1985). Therefore, it seems that the relatively older athletes have more opportunities to achieve a higher sports level, in terms of selection and competition performance, than their relatively younger peers (Till et al., 2010). Most of the explanations that have been provided in this regard have highlighted biological factors as the origin of the imbalance between athletes, focusing attention on anthropometric, physical, and physiological parameters (Baker et al., 2010). This “maturation-selection hypothesis” is the most argued and commonly cited theory (Helsen et al., 1998; Cobley et al., 2009), especially in team sports of a predominantly physical nature, to explain the advantages of relatively older players with regard to relatively young players. Other arguments displayed, primarily in team sports, are connected to the specific interactive factors of each sport exposed in the RAE constraints-based theoretical model (Wattie et al., 2015), so we could consider sociocultural factors (Wattie et al., 2008), geographic (Steingröver et al., 2017) and psychological criteria (Hancock et al., 2013), or linked to the competition itself (Yagüe et al., 2018), are some of the key factors that could modulate the impact of the RAE.

However, as the athlete ascends to higher performance levels (senior category), it does not seem so clear that relatively older athletes enjoy certain sport and competitive advantages over their younger peers (McCarthy and Collins, 2014). Thus, as the sports transition process progresses and especially in team sports such as football, it seems that the impact of the RAE tends to decrease but not disappear (Brustio et al., 2018; Gil et al., 2019). Furthermore, Gibbs et al. (2012) revealed how being an athlete born at the end of the year could be an advantage for the long-term sport development due to overcoming adversities and demands derived from the RAE— “underdog effect.” This possible circumstance, based on an overrepresentation of athletes who are relatively young or born in the last months of the year, is called RAE reversal (Cobley et al., 2009). Recently, there have been several investigations in team sports regarding this phenomenon, finding different explanations about its presence and magnitude. From a psychological perspective, it was found that relatively younger athletes presented, in the early stages of development, a psychological profile with a high degree of resilience (Collins and MacNamara, 2012; Sarkar et al., 2015). Other explanations were as follows: lower dropout rate by relatively young players due to a lower number of injuries than relatively older players (Bjørndal et al., 2018a), self-improvement experiences associated with adversity in selection processes (Collins and MacNamara, 2017), high levels of challenge in competition (McCarthy et al., 2016), or player recruitment systems (Sims and Addona, 2016). However, it should be clarified that the influence of the birthdate in professional sports (RAE vs. RAE reversal) does not yield results in the same direction, depending on factors connected to the sport context (Delorme et al., 2009; Lupo et al., 2019).

The RAE has been studied from a variety of approaches and with different purposes: to examine its presence in collective and individual sports contexts (Papadopoulou et al., 2019; Steidl-Müller et al., 2019; Mon-López et al., 2020), evaluate their influence on a fixed competition (Saavedra-García et al., 2019), check the degree of impact of gender and/or of age/competition categories in clubs or federal organizations (Bjørndal et al., 2018b; Romann et al., 2018), or even through intervention proposals the intention of which was to reduce the possible consequences (Mann and van Ginneken, 2017; Hill et al., 2019). Currently, it seems that the objective of the research is focused on studies whose aim is to analyze the relationship between the RAE and competition performance in order to know in-depth how the latter can be influenced by this phenomenon.

According to this approach, Singer and Janelle (1999) considered that the performance yielded in competition could serve as a useful tool to recognize sports excellence quantitatively. Although competition performance can be measured based on indicators of a different nature (i.e., biomechanical, technical, tactical, physiological, etc.), it is very common for team sports to often use clear, unequivocal, and useful indicators in relation to the successful result or not of the actions done and/or the matches played (Hughes and Bartlett, 2002). Analyzing these types of parameters, either in isolation or by comparison with other athletes or teams, an accurate measure of sport success could be obtained through indexing performance in team sports.

In the field of team sports, the analysis of sports success could be carried out on two levels: short-term and long-term performance. In the first kind of performance, it has normally been measured by weighing the collective results, using the final team position in the competition (McGarry, 2009); or through observation of individual statistical parameters (i.e., minutes played, goals scored, average performance indexes, etc.) that synthesize officially the participation, intervention, and performance of the players in competition (de la Rubia et al., 2020). Both indicators represent valuable information to accurately interpret the performance and interaction of the athlete with the environment, peers, and adversaries (Sampaio et al., 2015). At the same time, the growing interest in team sports to recognize the worth of the athletes individually has led to an increase in studies based on performance analysis through different personal attainments (salary, rankings, recognition, longevity, etc.) achieved throughout the athlete's sports career (Fumarco et al., 2017; Gil et al., 2019). This is the long-term consideration of competition performance.

The scientific literature presents, usually, the relationship between “RAE” and “performance” from a causal approach, that is, providing explanations why the birthdate could affect sport performance. Thus, the length of the performance period examined becomes a key factor. On the one hand, studies have been carried out with the aim to evaluate performance through a “cross-sectional analysis” based on determining sports success at a specific point in time. One of the main studies in this field (Vaeyens et al., 2005a,b) examined the performance variables “number of games played” and “time played,” that is, short-term statistical parameters. In this kind of studies, the impact and magnitude of the RAE can be accurately evaluated, but they have the inconvenience of assuming an equal distribution of the athletes by grouping method throughout the year (Dixon et al., 2020). On the other hand, studies based on the “longitudinal performance analysis” have proliferated with the aim of verifying the consequences derived from the RAE throughout the sport careers (Steingröver et al., 2016; Fumarco et al., 2017; Jones et al., 2018). The indicators most used to assess long-term performance have been individual or collective cumulative statistics, position in the ranking, victory rates, or number of national/international appearances. Through these investigations, it is possible to observe in depth the dissonances between the talent detection systems (clubs vs. federations), the influence of the RAE depending on the competition level (professional vs. amateur), or the dropout rate in a particular sporting context. However, these approaches often require differentiating overlapping parallel talent development processes that hinder a conclusion about the impact of RAE on long-term performance (Dixon et al., 2020).

The purpose of this study was to conduct a systematic review of the influence of RAE on competition performance in team sports at the national and international levels. Although the RAE has been studied in-depth in team sports, to the best of our knowledge, its influence on competition performance in team sports, measured both individually and collectively as well as in the short term and in the long term, is not exactly known. The literature published in this regard between January 2000 and December 2019 was examined with one main objective: (i) to analyze the influence of the RAE on competition performance according to the performance measurement indicators employed in each sample (type of result in competition and performance production period) based on the sample characteristics (gender and age group) and the sport context (type of sport, competition category, competition level, and competition period). Therefore, the importance of this study lies in the need to determine the impact of the RAE on the total sample of athletes and to synthesize the results derived from the relationship between RAE and competition performance in team sports, with the aim of questioning the convenience of talent detection models based on performance parameters in competition.

## Methods

### Study Design

The study design employed in this research was a systematic review with the aim of synthesizing the available scientific evidence through a qualitative review of the primary studies and summarizing the existing information (Manterola et al., 2013), in this case with regard to the influence of the RAE on competition performance in team sports. The stages of the systematic review procedure and subsequent qualitative and quantitative analysis of the scientific evidence adhered to the guidelines set out in the PRISMA (Preferred Reporting Items for Systematic Reviews and Meta-Analysis) checklist and the PICOS (Population, Interventions, Comparisons, Outcomes, and Study Design) question model for the definition of inclusion criteria.

### Participants—Inclusion and Exclusion Criteria

Original studies aimed at examining the relationship between the RAE and competition performance in team sports were included. Moreover, these studies could be published in peer-reviewed journals with an impact factor included in the Journal Citation Reports of the Web of Science (JCR of WoS) that were in English or Spanish language and in the period between the years 2000 and 2019 (previously, no significant relevant studies were found).

The inclusion criteria established for the systematic search, according to the PICOS question model, were as follows: (1) population: athletes with highest standard of performance in team sports belonging to the 1st competition level (top-tier professional leagues or tours—international level), 2nd level (2nd tier professional leagues or tours—national level), or 3rd level (athletes involved in talent development processes) and whose minimum level of sport success has taken place in a 2nd- or 3rd-level competition (Swann et al., 2015); (2) intervention: national and international official high-performance competitions with information about individual and/or collective performance; (3) comparison: relationship between individual and/or collective competition performance and athlete's birthdate within the same constituent year; (4) outcomes: competition performance according to two specific indicators, “type of result” (individual and/or collective) and “performance period” (short term and/or long term); (5) study design: observational-descriptive research based on establishing a relationship between the RAE and competition performance.

The following exclusion criteria were set: (1) studied the RAE in educational contexts (physical education or sport in the educational center); (2) evaluated the RAE in individual sports, in pairs or connected to refereeing; (3) showed a sample with competition levels below national and/or international; (4) carried out interventions on the ways of grouping the sample; (5) not provided data connected to the distribution of the participants according to the RAE; (6) exclusively examined other different results (acquisition skills, fitness, psychological, physical, and/or anthropometric tests); (7) examined cognitive performance; (8) exclusively determined a relationship between the RAE and performance in other terms (salary, market value, etc.); (9) examined relationships with other developmental and/or behavioral processes (leadership, anxiety, suicide, etc.); (10) analyzed, as a priority objective, interventions to solve the consequences derived from the RAE; (11) analyzed combined competition levels in the same sample (i.e., regional and national). In addition, those studies in editorial format, letter to the editor, comment, abstract, conference, or opinion article were excluded. Previously published systematic reviews about RAE in sport were only considered in order to find potentially valid studies for this scientific research.

### Search Strategy—Data Sources

The scientific studies compilation process was carried out through an exhaustive and systematic search in four electronic databases: Sport Discus, PubMed, Web of Science, and Scopus. The search terms used were grouped into three search strings: (1) “RAE” or “relative age” or “relative age effect^*^” or “influence of age” or “birthdate” or “birthdate effect^*^” or “age effect^*^” or “season of birth”; and (2) “American football” or “Australian football” or “baseball” or “basketball” or “cricket” or “football” or “futsal” or “handball” or “hockey” or “ice hockey” or “netball” or “rugby” or “soccer” or “softball” or “volleyball” or “team sport^*^” or “associative sport^*^”; and (3) “performance” or “minute^*^ played” or “game^*^ played” or “goal^*^” or “ranking” or “classification” or “place^*^” or “medal^*^” or “success” or “attainment” or “statistics.” Moreover, studies were incorporated through additional sources (bibliography of systematic reviews and alerts received by e-mail during the process).

### Systematic Review Protocol

**Figure 1:**
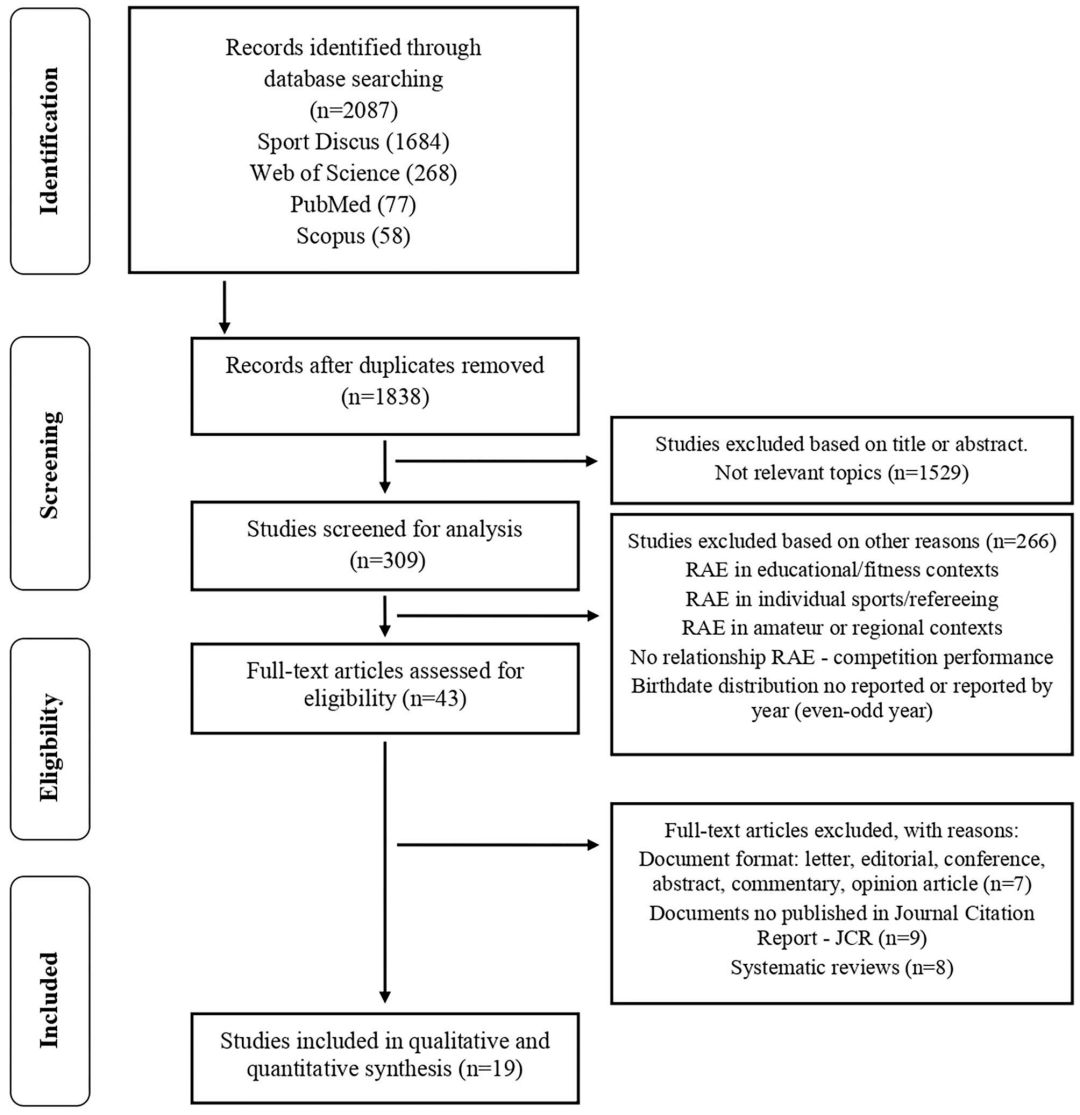
Flow diagram for screening and selection of studies according to Preferred Reporting Items for Systematic Reviews and Meta-Analysis (PRISMA).

To ensure the reliability of the search process and the suitable eligibility of scientific studies, the authors worked separately and independently. The process was carried out in the months of December 2019 and January 2020, and it was composed of the following phases (Figure 1), according to the criteria for preparing systematic reviews (PRISMA) (Liberati et al., 2009): (1) identification: the first author (A.R.) found 2,087 studies through the digital query of four databases (Sport Discus, PubMed, Web of Science, and Scopus) with the aim of increasing control over the reliability of the data associated with the existing scientific bibliography; (2) screening: the first author (A.R.) eliminated duplicate files (*n* = 249) and excluded those studied based on topics considered not relevant according to a previous reading of the title, abstract and keywords (n = 1,529). In addition, together with the second author (A.L.) and third author (J.L.), the first author (A.R.) rejected the studies about RAE contextualized in one of the following fields (*n* = 266): educational contexts, individual sports, refereeing, amateur, or local or regional competitions, with no link to sport performance and with no sample distribution by quartiles (Q1–Q4), semesters (S1–S2), or months of the year (M1–M12) as a function of the participants' birthdates. At the end of this stage, 43 studies were admitted by the authors; (3) eligibility: the first (A.R.), second (A.L.), and third authors (J.L.) eliminated full-text studies from the selection process by the following reasons: type of publication (*n* = 7), indexing of the journal (*n* = 9), or systematic review (*n* = 8); (4) inclusion: the remaining studies (*n* = 19) were finally considered for inclusion in the systematic review in order to analyze them, quantitatively and qualitatively, and synthesize the main results on the relationship between the RAE and competition performance.

### Data Collection and Extraction

All studies analyzed, read, and reviewed by the authors were published in English. In the data extraction process, the information was categorized according to the following items: (A) year of publication, (B) authors, (C) sample characteristics (number of athletes, gender, age, and age group), (D) sport context (type of sport, competition category, competition level, and competition period), (E) grouping method based on birthdate (month [M], quartile [Q], semester [S]), (F) competition performance indicators (in terms of result: individual and/or collective; in terms of performance production period: short term and/or long term), and (G) the relationship between the RAE and the competition performance (influence of the RAE, influence of the RAE reversal, or lack of influence).

### Analysis by Subcategories

In order to conduct an in-depth analysis of the impact of the RAE on competition performance according to established performance indicators, the sample of the studies under review was distributed into different subcategories according constraints-based theoretical model (Wattie et al., 2015). From each study, the data connected to the samples or set of athletes according personal characteristics and sport context (“n” and “%”), the athletes (“n” and “%”), and the relationship between the RAE and competition performance (“n” and “%”) were provided (absolute and relative frequency).

#### Sample Characteristics (Individual Constraints)

Regarding the characteristics of the sample (C), athletes were grouped according to (C1) “gender”: men and women, (C2) “age group”: adolescence (12–14 years), postadolescence (15–19 years), and adults (>19 years) (Baxter-Jones, 1995; World Health Organization, 2015; Smith et al., 2018). Samples composed of athletes from two different stages of human development (adolescence and postadolescence or postadolescence and adult) were registered as “not encodable.”

#### Sport Context (Task Constraints)

Based on the sport context (D), the athletes were assigned to the corresponding subcategory according to four items: (D1) “type of sport”: “invasion games” (American football, basketball, football/soccer, futsal, handball, ice hockey, rugby, and water polo) or “striking and fielding games” (baseball and cricket) (Read and Edwards, 1992); (D2) “competition category”: U-14, U-15, U-16, U-17, U-18, U-19, U-20, U-21, U-22, or senior; (D3) “competition level”: national or international [the samples composed of athletes who participated in several competition levels at the same year or season (i.e., regional and national level) were excluded].; (D4) “competition period”: prior to 2000, after 2000, or combined (beginning before 2000 and ending after 2000).

#### Grouping Method (Environmental Constraints)

Regarding the sample distribution and grouping method (E), the athletes were categorized according to the birthdate and cutoff date established for each sport and international and national federation So, the athletes were divided into annual or biannual competition cycle by (E1) “semesters”: semester 1/semester 3 (S1/S3) and semester 2/semester 4 (S2/S4); (E2) “quartiles”: quartile 1/quartile 5 (Q1/Q5), quartile 2/quartile 6 (Q2/Q6), quartile 3/quartile 7 (Q3/Q7), quartile 4/quartile 8 (Q4/Q8); (E3) “months”: month 1 (M1), month 2 (M2), month 3 (M3), month 4 (M4), month 5 (M5), month 6 (M6), month 7 (M7), month 8 (M8), month 9 (M9), month 10 (M10), month 11 (M11), month 12 (M12).

#### Sport Performance Indicators

Regarding the competition performance (F), the scientific evidence of the studies analyzed was registered according to two kinds of measurement indicators: (F1) “type of result” (individual or collective); (F2) performance production period (short term or long term). While the short-term performance refers to the statistical parameters associated with short competitions or regular seasons due to a presumable non-variation of the players of a team roster, the long term focuses on the evaluation of performance beyond a sport season or, even, throughout a sport career according to the statistical parameters accumulated individually and/or collectively. Combining both measurement criteria, the sample was categorized into four groups: short-term individual performance (individual statistics in competition), short-term collective performance (final team classification in competition), long-term individual performance (attainments throughout the sport career), and long-term collective performance (team rankings and maintenance period).

#### Influence of the RAE on Competition Performance

The samples were grouped by the influence of the RAE on competition performance (G). Thus, the athletes were included in one of the following groups: (G1) samples in which the RAE showed an impact on performance; (G2) samples in which the influence of RAE reversal on performance was detected; (G3) samples in which no relationship between the RAE and competition performance was identified.

### Study Quality Assessment

An adapted version according to “RAE–performance” Strengthening the Reporting of Observational Studies in Epidemiology (STROBE) checklist (Vandenbroucke et al., 2014; Smith et al., 2018) was employed to determine the quality of the studies object of the review (Table 1). The checklist constituted of 20 items grouped into six categories corresponding to the different sections of the study: “title-abstract” (item 1), “introduction” (items 2 and 3), “methods” (items 4–10), “results” (items 11–15), “discussion” (items 16–19), and “funding” (item 20). A score of 0 was awarded to the items with lack of information, and 1 to the items accurately described. The total score resulted from the addition of the item values, considering the following levels: “very low quality” (0–4 points), “low quality” (5–8 points), “medium quality” (9–12 points), “high quality” (13–16 points), and “very high quality” (17–20 points). Two independent reviewers (A.R. and A.L.) conducted study quality assessment. Rating disagreements were resolved by J.L., and interrater reliability calculated.

**Table 1:** Study quality assessment according to the Strengthening the Reporting of Observational Studies in Epidemiology (STROBE).

**References**	^*^1	^*^2	^*^3	^*^4	^*^5	^*^6	^*^7	^*^8	^*^9	^*^10	^*^11	^*^12	^*^13	^*^14	^*^15	^*^16	^*^17	^*^18	^*^19	^*^20	**Score**
Vaeyens et al. (2005a)	0	1	1	1	1	1	1	1	1	1	1	0	0	0	1	1	1	1	1	0	15
Vaeyens et al. (2005b)	0	1	1	1	1	1	1	1	1	1	1	0	0	0	1	1	1	1	1	0	15
Williams (2010)	0	0	0	1	0	1	1	1	0	1	1	0	0	0	1	1	1	1	0	0	10
Deaner et al. (2013)	0	1	0	1	1	0	1	1	1	1	0	1	1	1	1	1	1	1	0	0	14
García et al. (2014)	1	1	1	1	1	0	1	1	1	0	1	1	1	1	1	1	0	1	1	1	17
Karcher et al. (2014)	1	1	1	1	0	1	0	1	1	0	1	1	1	1	1	1	1	1	1	0	16
González-Víllora et al. (2015)	1	1	1	1	1	1	1	1	1	1	1	1	1	1	1	1	0	1	1	0	18
Arrieta et al. (2016)	0	0	1	1	0	1	1	1	1	0	1	1	0	0	1	1	0	0	1	1	12
Sims and Addona (2016)	0	1	1	1	0	1	1	1	1	1	1	1	1	0	1	1	0	0	1	1	15
Steingröver et al. (2016)	0	1	1	0	0	1	1	1	1	1	1	1	1	1	1	1	1	1	1	1	17
Torres-Unda et al. (2016)	0	1	1	1	1	0	0	1	1	1	1	0	0	0	1	1	1	1	0	1	13
Fumarco et al. (2017)	0	1	1	1	0	1	1	1	1	1	1	0	0	0	1	1	1	0	1	1	14
Rubajczyk et al. (2017)	1	1	1	1	1	1	1	1	1	1	1	1	1	1	1	1	1	1	0	1	19
Bjørndal et al. (2018a)	1	1	1	1	1	1	1	1	1	0	1	1	1	0	1	1	1	1	1	1	18
Ibañez et al. (2018)	1	1	1	1	1	1	1	1	1	1	1	1	1	0	1	1	1	1	1	1	19
Jones et al. (2018)	0	1	1	1	0	0	0	1	0	1	1	1	0	1	1	1	0	1	1	1	13
Yagüe et al. (2018)	0	0	1	1	0	1	1	1	1	1	1	1	1	1	1	1	0	1	1	0	15
Barrenetxea-Garcia et al. (2019)	0	0	0	1	0	1	1	1	0	0	1	1	1	1	1	0	1	1	0	1	12
Lago-Fuentes et al. (2019)	0	0	1	1	0	1	1	1	1	1	1	1	0	1	1	1	1	1	1	0	15

“*0” **=** item with absence or lack of information; “1” = item with complete and explicit information; In title and abstract,*

* 1 (title/abstract) = informative and balanced summary of what was done and what was found is provided. In introduction,

* 2 (background) = scientific background and rationale for the investigation being reported is explained;

* 3 (objectives) = state specific objectives and/or any pre-specified hypothesis. In Methods,

* 4 (setting) = setting, locations, and relevant dates for data collection are described. This must include information on study period (specific dates), sport context (type of sport, competition level, and competition category) and competition year(s) for all data collected;

* 5 (participants) = give characteristics of the sample (overall number, age, gender);

* 6 (participants) = procedure for selecting and grouping athletes in the sport context under evaluation (i.e., through cutoff date based on birthdate) and the way grouping according study purposes (i.e., by quartiles) are described;

* 7 (data source) = source and procedure for obtaining the birthdate and performance characteristics of the sample (RAE and individual and collective performance statistics) are described;

* 8 (data source) = procedure for determining performance measurement (individual and/or collective) is described;

* 9 (statistical methods) = statistical methods, including specific analytical methods used to examine subgroups and interactions (relationship between RAE and performance), are described;

* 10 (statistical methods) = how duplicates and missing data were addressed or incomplete data were handled (if applicable) is explained in results,

* 11 (descriptive results) = the number (absolute frequency) or percentage (relative frequency) of participants found in each grouping category and subcategory are reported;

* 12 (main results) = statistical estimate and precision (i.e., 95% CI) for each sample or subgroup group examined is provided;

* 13 (main results) = post hoc comparisons (OR) between grouping category (i.e., Q1 vs. Q4) are provided when appropriate;

* 14 (main results) = a measure of effect size is provided (i.e., Cramer's V, phi coefficient, Cohen's);

* 15 (main results) = a coefficient of correlation between RAE and performance measures is provided. In Discussion,

* 16 (key results) = a summary of key results with reference to study objectives is provided;

* 17 (limitations) = limitations of the study, taking into account sources of potential bias or imprecision are discussed;

* 18 (interpretation) = a cautious overall interpretation of results considering objectives and relevant evidence is provided;

* 19 (generalizability) = the generalizability of the study results to similar or other contexts is provided. In Funding,

* *20 (funding) = the funding source of the study is cited or the absence of funding, if applicable*.

## Results

### Qualitative Analysis—Study Selection and Characteristics

The quality analysis (RAE–performance STROBE checklist) yielded the following results (Table 1, Annex 1). Of the 19 included investigations, 15.79% (*n* = 3) were considered to “medium quality” (9–12 points); 52.63% (*n* = 10) were categorized as “high quality” (13–16 points); and 31.58% (*n* = 6) were considered to “very high quality” (17–20 points). The quality scores of the studies were found between the values 10 (lower limit) and 19 (upper limit), so that no article was classified as “very low quality” (0–4 points) or “low quality” (5–8 points). The average score of the studies analyzed was 15.11 points.

According to the analysis by sections, it was observed that the highest scores were located in “Introduction” (78.95%), “Methods” (80.45%), “Results” (75.79%), and “Discussion” (80.26%). Among the highest quality studies, item 8 (“Data source—procedure for determining performance measurement”) and item 15 (“Main results—a coefficient of correlation between RAE and performance measures”) were considered complete in all cases (100%), whereas the most commonly absent or incomplete items (0 points) were found in item 5 [“Participants—sample characteristics” (47.37%)], item 14 (“Main results—a measure of effect size” (52.63%)], and item 13 [“Main results—*post-hoc* comparisons between the different categories/groups” (57.89%)]. The lowest scores were found in “Abstract” (68.42%) and “Funding” (42.11%) sections.

### Quantitative Analysis

The quantitative analysis was based on the examination and evaluation of the RAE on the sample universe registered in the different studies of the review, according to the main characteristics of the athletes (individual constraints) and the sport context provided for each group of athletes (task constraints), as well as the relationship that the RAE presents with competition performance, depending on the different performance indicators used. Scientific evidence and detailed summary were included.

### Sample Characteristics (Individual Constraints) and Sport Context (Task Constraints)

#### Scientific Evidence

Scientific evidence of the analyzed and reviewed studies on the characteristics of the sample (C) and the sport context (D) is shown in Table 2. Format and design, including the title, the author, and the year of publication; the sample characteristics (overall number, gender, age); and the characteristics of the sport context (type of sport, competition category, competition level, and competition period) were included. The studies are arranged chronologically to favor an interpretation and longitudinal evaluation of the findings.

**Table 2:** Distribution of the sample according to the characteristics of the athletes (*n*, age and gender), sport context (type, competition category, competition level and competition period), grouping method (months [M], quartiles [Q], semesters [S]) and its impact on the set of birthdates (relative age effect).

**Author(s)**	**Sample characteristics**			**Sport context**				**Grouping method**	**Relative age effect**
	**N**	**Age**	**Gender**	**Type of sport**	**Competition category**	**Competition** **level**	**Competition period**		
Vaeyens et al. (2005a)	1,559	<21	M	Soccer (IG)	Senior	Belgian Football League (2nd and 3rd division) → NL	1998–2002	By quartiles (Q1–Q4)	RAE
	2,069	>21	M	Soccer (IG)	Senior		1998–2002		RAE
Vaeyens et al. (2005b)	1,640	16–39 (←1980)	M	Soccer (IG)	Senior	Belgian Football League (2nd and 3rd division) → NL	1998–2002	By months (M1–M12)	RAE
	498	16–39 ( → 1980)	M	Soccer (IG)	Senior		1998–2002		RAE
Williams (2010)	288	16–17	M	Soccer (IG)	U-17	FIFA Football World Cup → IL	1997	By months (M1–M12)	RAE
	288	16–17	M	Soccer (IG)	U-17		1999		RAE
	288	16–17	M	Soccer (IG)	U-17		2001		RAE
	320	16–17	M	Soccer (IG)	U-17		2003		RAE
	320	16–17	M	Soccer (IG)	U-17		2005		RAE
	480	16–17	M	Soccer (IG)	U-17		2007		RAE
Deaner et al. (2013)	8186	—	M	Ice Hockey (IG)	U-21	National Hockey League (NHL) → NL	1980–2012	By quartiles (Q1–Q4)	RAE R
García et al. (2014)	143	16–17	M	Basketball (IG)	U-17	FIBA Basketball WorldChampionship → IL	2010	By quartiles (Q1–Q4)	RAE
	191	18–19	M	Basketball (IG)	U-19		2011		RAE
	138	20–21	M	Basketball (IG)	U-21		2005		No RAE
	144	16–17	F	Basketball (IG)	U-17		2010		RAE
	194	18–19	F	Basketball (IG)	U-19		2011		RAE
	144	20–21	F	Basketball (IG)	U-21		2007		No RAE
Karcher et al. (2014)	192	20–41	M	Handball (IG)	Senior	Olympic Games London 2012	2012	By quartiles (Q1–Q8) By semesters (S1–S4)	RAE
	128	20–41	M	Handball (IG)	Senior	Handball World Championship	2013		RAE
	192	20–41	M	Handball (IG)	Senior	Handball European Championship → IL	2014		RAE
González-Víllora et al. (2015)	145	16–17	M	Soccer (IG)	U-17	UEFA European Soccer Championship → IL	2012	By quartiles (Q1–Q4) By semesters (S1–S2)	RAE
	144	18–19	M	Soccer (IG)	U-19		2012		RAE
	184	20–21	M	Soccer (IG)	U-21		2011		RAE
	368	22 →	M	Soccer (IG)	Senior		2012		No RAE
Arrieta et al. (2016)	455	15–16	M	Basketball (IG)	U-16	FIBA European Basketball Championship → IL	2013	By quartiles (Q1–Q4)	RAE
	454	17–18	M	Basketball (IG)	U-18		2013		RAE
	384	19–20	M	Basketball (IG)	U-20		2013		RAE
	396	15–16	F	Basketball (IG)	U-16		2013		RAE
	407	17–18	F	Basketball (IG)	U-18		2013		RAE
	299	19–20	F	Basketball (IG)	U-20		2013		No RAE
Sims and Addona (2016)	30,200	16–18	M	Baseball (SFG)	Senior	Major League Baseball (MLB) → NL	1987–2011	By months (M1–M12)	RAE R
Steingröver et al. (2016)	407	16–18	M	Basketball (IG)	Senior	National Basketball Association (NBA)	1980–1989	By quartiles (Q1–Q4)	No RAE
	1,028	16–18	M	Ice Hockey (IG)	Senior	National Hockey League (NHL)	1980–1989		RAE
	2,380	16–18	M	American Football (IG)	Senior	National Football League (NFL) → NL	1980–1989		No RAE
Torres-Unda et al. (2016)	72	13–14	M	Basketball (IG)	U-14	ACB—Mini Cup of Spain → NL	2010	By quartiles (Q1–Q4)	RAE
Fumarco et al. (2017)	2,363	18	M	Ice Hockey (IG)	Senior	National Hockey League (NHL) → NL	2008–2016	By quartiles (Q1–Q4)	RAE R
	1,538	19	M	Ice Hockey (IG)	Senior		2008–2016		RAE R
	546	20	M	Ice Hockey (IG)	Senior		2008–2016		RAE R
Rubajczyk et al. (2017)	1,223	13–14	M	Basketball (IG)	U-14	Polish Youth Basketball Championships (PZK) → NL	2013–2016	By quartiles (Q1–Q4) By semesters (S1–S2)	RAE
	927	15–16	M	Basketball (IG)	U-16		2013–2016		RAE
	907	17–18	M	Basketball (IG)	U-18		2013–2016		RAE
	792	19–20	M	Basketball (IG)	U-20		2013–2016		RAE
	1,228	13–14	F	Basketball (IG)	U-14		2013–2016		RAE
	922	15–16	F	Basketball (IG)	U-16		2013–2016		RAE
	900	17–18	F	Basketball (IG)	U-18		2013–2016		RAE
	369	19–22	F	Basketball (IG)	U-22		2013–2016		RAE
Bjørndal et al. (2018a)	299	18–19	M	Handball (IG)	U-19	Norwegian Handball Federation (NHF) → NL	2016–2017	By quartiles (Q1–Q8)	RAE
	182	20–21	M	Handball (IG)	U-21		2016–2017		RAE
	55	21 →	M	Handball (IG)	Senior		2016–2017		No RAE
	256	17–18	F	Handball (IG)	U-18		2016–2017		RAE
	190	19–20	F	Handball (IG)	U-20		2016–2017		RAE
	45	20 →	F	Handball (IG)	Senior		2016–2017		No RAE
Ibañez et al. (2018)	334 247	17–18 17–18	M M	Basketball (IG) Basketball (IG)	U-18 U-18	Adidas N. Generation Tournament → IL	2013–2014 2014–2015	By quartiles (Q1–Q4) By semesters (S1–S2)	RAE RAE
Jones et al. (2018)	262	—	M	Cricket (SFG)	Senior	International Cricket Council (ICC)	1994–2004	By quartiles (Q1–Q4)	RAE
	690	—	M	Rugby (IG)	Senior	International Rugby U. Players (IRUP) → IL	1994–2004		RAE R
Yagüe et al. (2018)	523	—	M	Soccer (IG)	Senior	L. Santander (Spain)	2016–2017	By quartiles (Q1–Q4) By semesters (S1–S2)	RAE
	596	—	M	Soccer (IG)	Senior	Ligue 1 (France)	2016–2017		RAE
	543	—	M	Soccer (IG)	Senior	Bundesliga (Germany)	2016–2017		RAE
	573	—	M	Soccer (IG)	Senior	Premier (England)	2016–2017		RAE
	632	—	M	Soccer (IG)	Senior	Serie A (Italy)	2016–2017		RAE
	450	—	M	Soccer (IG)	Senior	Eerste Klasse (Belgium)	2016–2017		No RAE
	522	—	M	Soccer (IG)	Senior	SüperLig (Turkey)	2016–2017		RAE
	297	—	M	Soccer (IG)	Senior	Bundesliga (Austria)	2016–2017		RAE
	521	—	M	Soccer (IG)	Senior	Eredivisie (Netherlands)	2016–2017		RAE
	544	—	M	Soccer (IG)	Senior	Primeira Liga (Portugal)	2016–2017		RAE
						→ NL			
Barrenetxea-Garcia et al. (2019)	622	—	M	Water polo (IG)	Senior	2011, 2013 and 2015 World Water Polo Championships → IL	2011–2015	By quartiles (Q1–Q4)	No RAE
	623	—	F	Water polo (IG)	Senior		2011–2015		No RAE
Lago-Fuentes et al. (2019)	183	—	M	Futsal (IG)	Senior	National League of Futsal (LNFS, Spain) → NL	2006–2007	By quartiles (Q1–Q4)	RAE R
	206	—	M	Futsal (IG)	Senior		2007–2008		RAE R
	201	—	M	Futsal (IG)	Senior		2008–2009		RAE R
	205	—	M	Futsal (IG)	Senior		2009–2010		RAE R
	219	—	M	Futsal (IG)	Senior		2010–2011		RAE R
	218	—	M	Futsal (IG)	Senior		2011–2012		RAE R
	203	—	M	Futsal (IG)	Senior		2012–2013		RAE R
	211	—	M	Futsal (IG)	Senior		2013–2014		RAE R
	227	—	M	Futsal (IG)	Senior		2014–2015		RAE R

*N, absolute frequency of the sample; “←”, before; “ → ”, after; “—”, information not provided; M, male; F, female; U-14, under 14; U-16, under 16; U-17, under 17; U-18, under 18; U-19, under 19; U-20, under 20; U-21, under 21; U-22, under 22; IG, invasion games; SFG, striking and fielding games; NL, national level; IL, international level; M1-M12, birth month; Q1-Q4/Q8, birth quarter; S1-S2/S4, birth semester; no RAE, no relative age effect; RAE, relative age effect; RAE R, relative age effect reversal*.

#### Summary

Table 3 shows a synthesis of the scientific evidence of the sample according to constraints-based theoretical model based on the main characteristics of the participants (gender, age, and age group) and the sport context (type of sport, competition category, competition level, and competition period). Through the analysis of the 19 scientific studies (C), 77 independent samples composed of 77,329 athletes were identified. Examining the sample characteristics (individual constraints): (1) depending on the “gender,” the sample distribution was biased because of 92.08% of the athletes being men (*n* = 71,202), whereas only 7.92% were women (*n* = 6,117); (2) regarding the “age” of the athletes, the sample set was identified as between 13 (lower limit) and 41 years old (upper limit). However, no data were found connected to the age of the participants in 24 samples (31.17%), recording 17,457 athletes with no identification by age. Thus, the athletes were unevenly distributed according to the “age group”: adolescence [*n* = 2,523 (3.26%)], postadolescence [*n* = 18,446 (23.85%)], and adults [*n* = 46,026 (59.52%)]. Of the total number of players, 13.35% (*n* = 10,324) could not be categorized because the sample crossed two or more different age groups without determining the exact age.

**Table 3:** Summary of samples (*n*) and athletes (*n* and %) by characteristics of athletes (gender and age group) and sport context (type of sport, competition category, competition level and competition period).

**Category**	**Subgroup category**	**Samples (*n*)**	**Athletes, n (%)**
Gender	Male	63	71,202 (92.08)
	Female	14	6,117 (7,92)
Age group	Adolescence (12–14)	3	2,523 (3.26)
	Postadolescence (15–19)	32	18,446 (23.86)
	Adult (>19)	39	46,026 (59.53)
	Not encodable	3	10,324 (13.35)
Type of sport	Invasion games	75	46,867 (60.61)
	Striking and fielding games	2	30,462 (39.39)
Competition category	U-14	3	2,523 (3.26)
	U-16	4	2,700 (3.49)
	U-17	9	2,416 (3.12)
	U-18	7	3,505 (4.53)
	U-19	4	828 (1.07)
	U-20	4	1,665 (2.15)
	U-21	5	8,834 (11.43)
	U-22	1	369 (0.48)
	Senior	40	54,479 (70.46)
Competition level	National	40	66,828 (86.42)
	International	37	10,491 (13.58)
Competition period	← 2000	5	5,343 (6.91)
	2000 →	61	27,824 (35.99)
	← 2000 →	11	44,152 (57.10)

*n, absolute frequency; %, relative frequency; U-14, under 14; U-16, under 16; U-17, under 17; U-18, under 18; U-19, under 19; U-20, under 20; U-21, under 21; U-22, under 22; ← 2000, prior to year 2000; 2000 → , after the year 2000; ← 2000 → , prior and after to year 2000*.

According to the sport context (task constraints) (D), the following results by subcategory were observed: (1) based on the “type of sport,” most of the samples analyzed (*n* = 75) corresponded to sports called “invasion games,” adding a total of 46,867 athletes (60.61%), whereas only two samples (baseball and cricket) belonged to the so-called “striking and fielding games” with 30,462 athletes (39.39%). (2) In relation to the “competition category,” the most evaluated development stage was “senior” with 54,479 athletes (70.45%) distributed in 40 samples. In the other categories, the remaining 37 samples were recorded, collecting the following values in terms of number of athletes: U-14 (*n* = 2,523), U-16 (*n* = 2,700), U-17 (*n* = 2,416), U-18 (*n* = 3,505), U-19 (*n* = 828), U-20 (*n* = 1,665), U-21 (*n* = 8,834), and U-22 (*n* = 369). (3) According to the “competition level,” the performance of 13.58% of the athletes was examined in international competitions (*n* = 10,491), such as World Championships or Olympic Games, whereas the performance evaluation of 86.42% of the athletes was carried out in national contexts of competition (*n* = 66,828), such as Leagues or Cups of the respective countries. (4) Finally, regarding the “competition period,” 57.10% of the athletes analyzed (*n* = 44,152) were part of longitudinal studies that combined in their analysis previous and post-2000 seasons. The studies carried out, entirely, from the year 2000 represented, in terms of the number of athletes, 35.98% (*n* = 27,824), whereas those that were carried out before the year 2000 were 6.91% (*n* = 5,343).

### Sample Distribution (Environmental Constraints)

#### Scientific Evidence

Considering the criteria for grouping athletes (E) according to chronological age and the cutoff date, the scientific evidence of the results is shown in Table 2. Format and design, including the title, the author, and the year of publication; the overall number of the participants; the grouping method (months, quartiles, or quartiles/semesters); and type of distribution of the participants (RAE, RAE reversal, or no RAE) were included. The studies are arranged chronologically to favor an interpretation and longitudinal evaluation of the findings.

#### Summary

**Figure 2:**
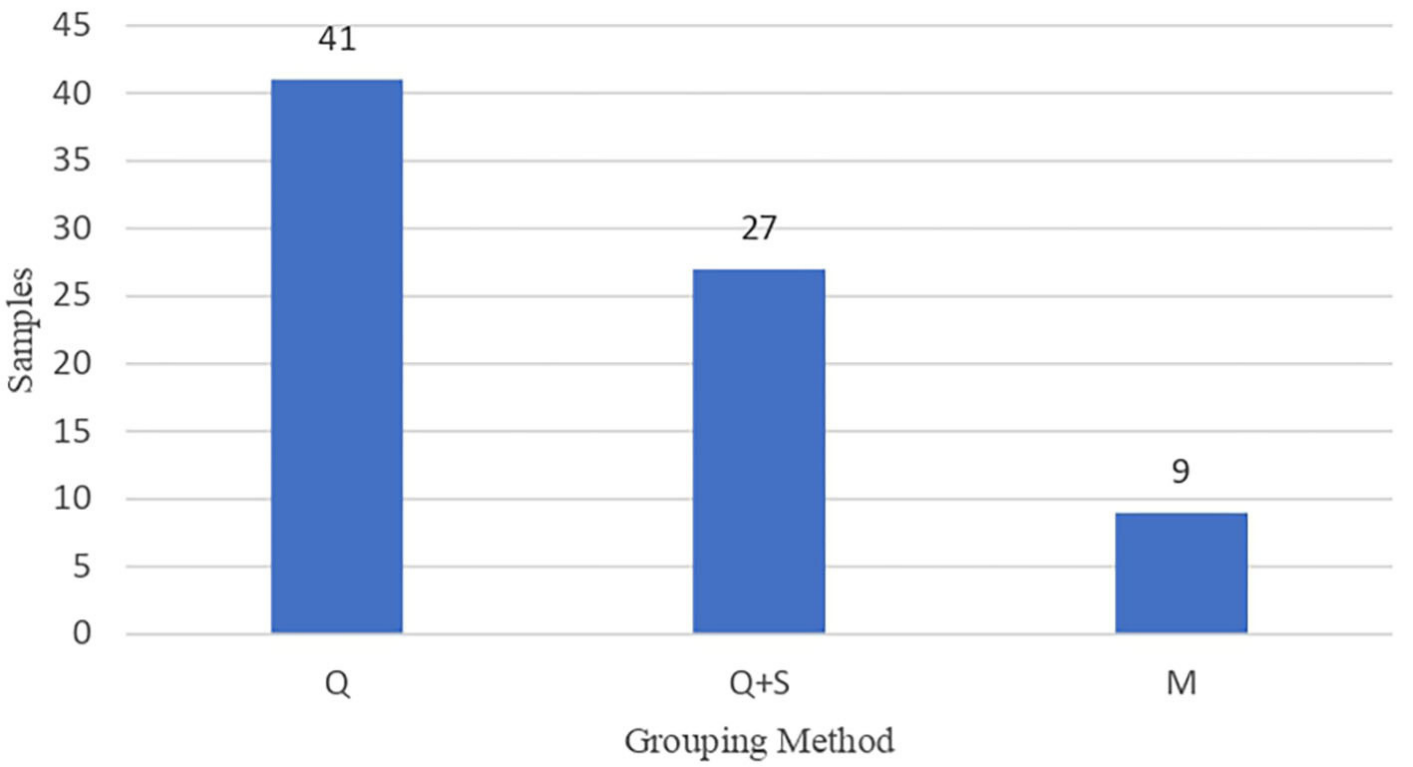
Distribution of the samples (N) according to the grouping method (quartiles [Q]; quartiles and semesters [Q + S] and months [M]).

**Figure 3:**
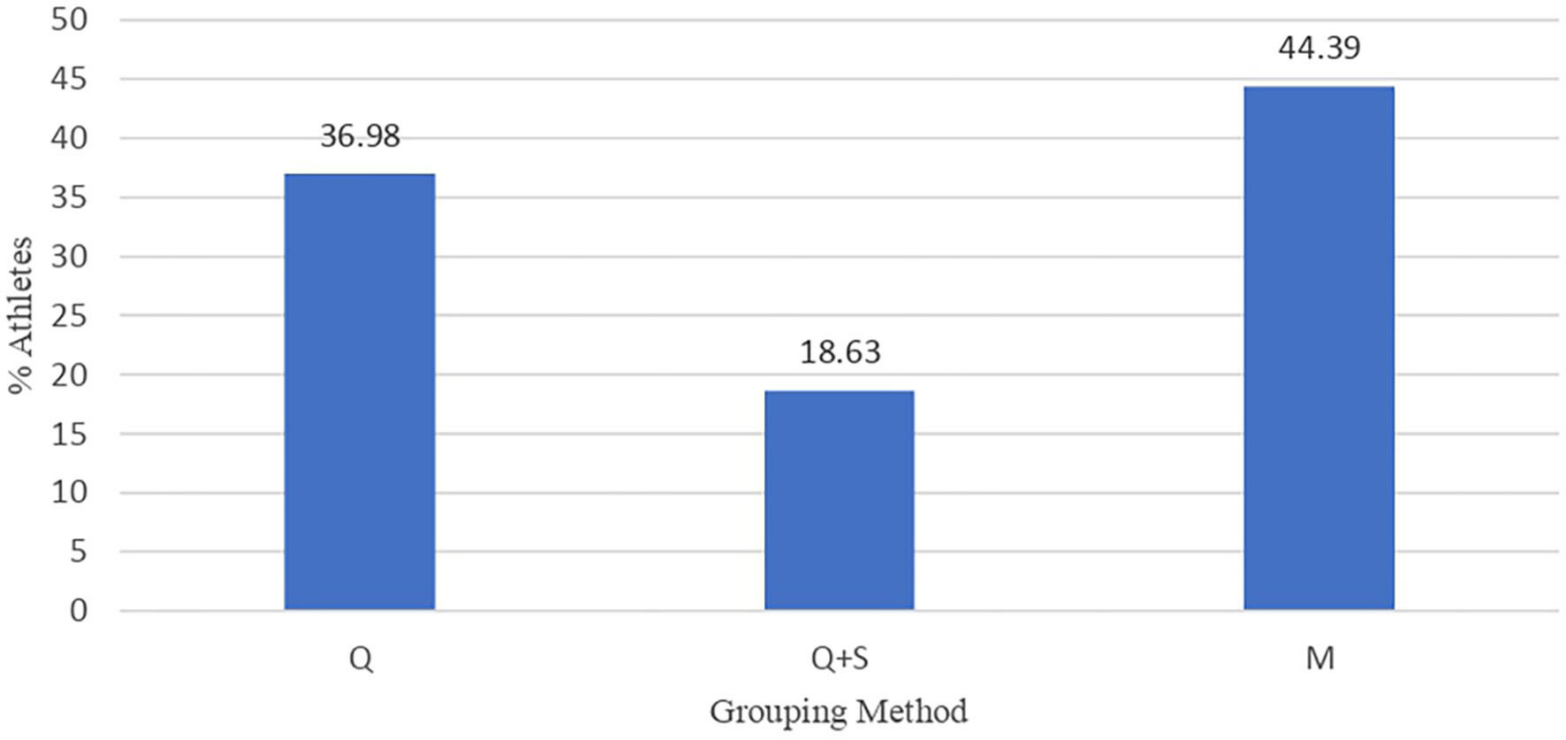
Distribution of the athletes (%) according to the grouping method (quartiles [Q];quartiles and semesters [Q + S] and months [M]).

The sample distribution and the athletes that composed it are shown in Figures 2, 3. The most used method for the grouping of athletes was, according to number of samples, the birthdate considering the year by quartiles [41 samples (28,594 athletes)]. Among these samples, the distribution by quartiles was not the same if the competition period was considered as an annual cycle made up of four quartiles and two semesters (87.5%) or a biannual cycle made up of eight quartiles and four semesters (12.5%). In another 27 samples, 14,403 athletes were categorized, in addition to quartiles, by semesters. In the other samples (*n* = 9), the grouping method according to the natural month of birth was identified, accounting, surprisingly, for 34,322 athletes.

Regarding the number of registered athletes according to the grouping method (quartiles, “Q”; semesters, “S”; months, “M”), an unequal and biased distribution was observed in 66 samples (71,788 athletes). Among them, RAE was detected in 51 samples and 26,392 athletes (34.12%), whereas the RAE reversal was found in 15 samples and 45,396 athletes (58.71%). In the remaining 10 samples, no impact of the RAE on a representation of 5,531 athletes (7.17%) was identified.

Taking as a reference the set of athletes in whom the RAE (RAE or RAE reversal) was detected [*n* = 71,788 (92.83%)], a summary based on the characteristics of the athletes (individual constraints) and the sport context (task constraints) is included in Table 4. With regard to the sample characteristics, the RAE did not have the same impact. Considering the sample characteristics: (1) according to “gender,” an overrepresentation of relatively young athletes in male distributions was observed, accounting for 45,396 athletes (63.25%), whereas biased distributions in favor of relatively older players grouped 21,386 athletes (29.79%). On the other hand, in most of the samples analyzed in women's sports, a distribution with an overrepresentation of relatively older players was identified [*n* = 5,006 (6.97%)], with no cases of RAE reversal being recorded. (2) Assessing the “age group,” the RAE reversal was identified in 60.54% of the samples, with more frequency in the adult age group (>19 years old) where 33,309 athletes were registered, whereas in the immediately lower development stage, postadolescence (15–19 years old), the number of athletes was considerably less (*n* = 3,901). No cases of RAE reversal were found in adolescent athletes (12–14 years old). With regard to the analyzed samples that showed a selection bias favorable to relatively older athletes (39.46%), the results confirmed a higher impact of the RAE in the adult age (*n* = 10,272) and postadolescence (*n* = 11,459) than in the adolescent age (*n* = 2,523).

**Table 4:** Summary of sample's distribution (*n* and %) according to the relative age effect identified (RAE or RAE reversal) by characteristics of athletes (gender and age group) and sport context (type of sport, competition category, competition level, and competition period).

		**RAE**		**RAE reversal**	
**Category**	**Subgroup category**	**Samples**	**Athletes**	**Samples**	**Athletes**
		***n***	***n* (%)**	***n***	***n* (%)**
Sample characteristics	Gender				
	Male	41	21,386 (29.79)	15	45,396 (63.24)
	Female	10	5,006 (6.97)	0	0 (0)
	Age group*				
	Adolescence (12–14)	3	2,523 (4.11)	0	0 (0)
	Postadolescence (15–19)	27	11,459 (18.64)	2	3,901 (6.35)
	Adult (>19)	19	10,272 (16.71)	12	33,309 (54.19)
Sport context	Type of sport				
	Invasion games	50	26,130 (36.40)	14	15,196 (21.17)
	Striking-fielding games	1	262 (0.36)	1	30,200 (42.07)
	Competition category				
	U-14	3	2,523 (3.52)	0	0 (0)
	U-16	4	2,700 (3.76)	0	0 (0)
	U-17	9	2,416 (3.37)	0	0 (0)
	U-18	7	3,505 (4.88)	0	0 (0)
	U-19	4	828 (1.15)	0	0 (0)
	U-20	3	1,366 (1.90)	0	0 (0)
	U-21	2	366 (0.51)	1	8,186 (11.41)
	U-22	1	369 (0.51)	0	0 (0)
	Senior	17	12,319 (17.16)	14	37,210 (51.83)
	Competition level				
	National	23	18,885 (26.31)	14	44,706 (62.28)
	International	28	7,507 (10.46)	1	690 (0.96)
	Competition period				
	←2000	4	1,866 (2.60)	1	690 (0.96)
	2000 →	43	18,760 (26.14)	12	6,320 (8.80)
	←2000 →	4	5,766 (8.03)	2	38,386 (53.47)

n, absolute frequency; %, relative frequency; U-14, under 14; U-16, under 16; U-17, under 17; U-18, under 18; U-19, under 19; U-20, under 20; U-21, under 21; U-22, under 22; ←2000, prior to year 2000; 2000 → , after the year 2000; ←2000 → , prior and after to year 2000. *Because of the lack of data about the age of participants in some studies, the analysis of the sample by age group was reduced to 61,464 athletes.

Considering the sport context, the sample also presented an unequal and biased distribution of the athletes. (1) Based on the “type of sport,” the RAE reversal was mainly detected in the “striking and fielding games,” which affected 30,200 athletes, whereas in 262 of them, the presence of RAE was observed. In the “invasion games,” the trend was reversed showing a greater weight of the influence of the RAE on the athletes (*n* = 26,130) than the RAE reversal (*n* = 15,196). (2) According to the “competition category,” in the athlete's formative ages, a prevalence of the samples in which the selection process to participate in official competitions was biased in favor of relatively older players was observed (U-14: *n* = 3; U-16: *n* = 4; U-17: *n* = 9; U-18: *n* = 7; U-19: *n* = 4; U-20: *n* = 3; U-21: *n* = 2; U-22: *n* = 1). In contrast, only one biased sample was identified in favor of relatively young players (U-21: *n* = 1). Similar results were found in the senior category, where the samples in which RAE was identified (*n* = 17) continued to be larger than the samples showing an RAE reversal (*n* = 14). However, by number of athletes, the connection is reversed at a ratio of 3:1 (RAE: *n* = 12,319; RAE reversal: *n* = 37,210). (3) Based on the “competition level,” a notable presence of the RAE reversal was observed in the samples of national competitions (62.28%) including 44,706 athletes, whereas in international competitions, this presence was minimal [*n* = 690 (0.96%)]. However, in the cases with the presence of RAE, large differences were not found because of 18,885 athletes (26.31%) being identified in national contexts, whereas 7,507 athletes (10.48%) were detected in international contexts. (4) Finally, regarding the “competition period,” the RAE reversal affected more than half of the athletes in the whole sample [*n* = 38,386 (53.47%)] whose performance was analyzed before and after the year 2000, whereas the RAE was identified in 18,760 athletes (26.13%) from samples of studies after the year 2000. In the investigations carried out before 2000, the difference did not seem to be highlighted because of a considerable number of samples not being found (*n* = 5).

### Relationship Between RAE and Competition Performance

#### Scientific Evidence

The synthesis of the relationship between RAE and competition performance in team sports (G) based on performance indicators (F) is shown in Table 5. Format and design, including the title, the author, and the year of publication; the aim of the study; the indicators used for measuring the performance in competition; the main results of the investigation associated with the relationship between RAE and performance in competition; and the most relevant conclusions were included. The studies are presented in chronological order to emphasize their longitudinal interpretation.

**Table 5:** Relationship between RAE and competition performance providing aim of the study, performance indicators, main results and conclusions.

**References**	**Aim of the study**	**Performance indicators**	**Main results (RAE–performance)**	**Conclusions**
Vaeyens et al. (2005a)	(1) To examine whether semiprofessional and amateur soccer teams complied with the under-21 rule (S1); and (2) to determine if the under-21 rule was effective in increasing the playing opportunities of young adult (under-21) soccer players (S2)	Individual statistics:no. of games played and time played (min)	1. Relatively older amateur or semiprofessional players in the senior category (>21 years old) played a higher number of minutes and they were selected a bigger number of appearances than relatively young players 2. Relatively young players (U-21) were selected to participate with the first teams from the bench and for short periods of time	Influence of RAE on short-term individual performance
Vaeyens et al. (2005b)	The present study had two objectives: (1) to compare the relative age (RA) effect in players before and after the change in cutoff for the selection year (1997). And (2) to use match-related variables in addition to birth dates to examine the RA	Individual statistics:no. of games played and time played (min)	1. Competition performance, measured in games and minutes played, was higher in relatively older players, both in the G1B (cutoff date on August 1; born before 1980) and in the G2B (cutoff date on January 1; born after 1980)	Influence of RAE on short-term individual performance
Williams (2010)	This investigation sought to determine if a RA effect exists in the FIFA U-17 World Cup competition	Collective statistics:final team position	1. The performance, according to the final team position in each of the World Soccer Championships analyzed, was larger in those teams that had a higher percentage of relatively older players or early maturing players in their squads	Influence of RAE on short-term collective performance
Deaner et al. (2013)	(1) Our analyses of productivity included all selections from all draft rounds for a period of 27 years; (2) we tested whether birth quarter was associated with productivity once draft slot was controlled; (3) we investigated a potential mediator of selection bias, the decision to become draft eligible; (4) we tested for changes in selection bias over time; (5) we examined whether selection bias reduces relatively younger individuals' playing opportunities	Individual statistics throughout the sports career: no. of games; goals per game; assists per game; pints per game (goals + assists); offensive and defensive productivity measure	1. The relatively young players chosen in the last rounds of the draft (+101st round) achieved better competition performance, in terms of games played and points per game, than the relatively older ones. Attenuated results were observed in the drafted players between rounds 1st and 100th 2. The relatively young players, chosen within the first year of the draft (18 years old), achieved longer competition performance indicators (games played and points scored) than relatively young players chosen in the second year (19 years old) and third year (20 years old) respectively 3. Relatively young players performed better, throughout their sports careers, than relatively older players	Influence of RAE reversal on long-term individual performance
García et al. (2014)	To check whether the RA effect does exist in the World Basketball Championship U-17, U-19, and U-21 male and female categories, to investigate if the RA effect exists in the different specific positions and also try to find differences in height and in performance between players depending on their birthdate	Individual statistics:games played; minutes played; converted field goals (% effectiveness); 2-point field goals (% effectiveness); 3-point field goals (% effectiveness); free goals scored (% effectiveness); def. rebounds; off. Rebounds; assistances; personal faults; stolen; recuperations; blocked; points; points per game	1. Relatively older players performed better on the following statistical parameters: 3-point % (male U-17); points per game (male U-19); assists and assists per game (female U-19) 2. In contrast, relatively young players performed better on the following statistical parameters: 2-point % and free-throw % (female U-19) 3. However, could be not affirmed, in general, that the competition performance in basketball, measured in statistical terms, was affected by the RAE	No relationship between RAE and short-term individual performance
Karcher et al. (2014)	To examine the effects of month and year of birth on playing time during international competitions with respect to playing positions	Individual statistics:time played (min)	1. The probability of playing more than 50% of the time competition tended to be higher in relatively older players than relatively young players. However, no significant impact of the RAE, expressed in quartile (Q) and/or semester (S), was observed on playing time	No relationship between RAE and short-term individual performance
González-Víllora et al. (2015)	To examine the birthdates of the international players, together with other variables in the 2012 European Soccer Championship at a senior level and in U-21, U-19, and U-17 from the previous European Soccer Championship	Collective statistics:final team position	1. An overrepresentation of relatively older players was detected in those teams (U-17, U-19, and U-21 category) that achieved high performance in competition according to their classification (quarterfinals, semifinalists, finalists, and champion) 2. No such phenomenon was observed in the senior category	Influence of RAE on short-term collective performance(U-17, U-19 y U-21) No relationship RAE -performance (senior)
Arrieta et al. (2016)	To analyze the presence of the RAE and the possible relation of RA with performance in male and female European Youth Basketball Championships	Individual statistics:minutes, points, assists, steals, blocked shots, rebounds, personal fouls, missed shots, turnovers, personal, PIRCollective statistics:final team position	1. Relatively older players obtained higher individual performance indicators, in absolute and weighted terms, and collective performance according to final team position in competition than relatively young players in the U-20 category. The impact was less in U-16 and U-18 2. In women, the relationship between RAE and performance lost significance when the results were weighted for minutes played	Influence of RAE on short-term individual and collective performance (men)No relationship RAE -performance (women)
Sims and Addona (2016)	We explore the relationships of age and RA for players drafted out of HS with baseball career performance, using four different performance metrics (whether or not the player reached the major leagues, games played in MLB, career wins above a replacement player (WAR), and career on-base plus slugging percentage (OPS) for non-pitchers	Individual statistics throughout the sports career: time played (min); measure the additional number of wins that the player's team accumulates over a replacement-level player; running average	1. Relatively young drafted players achieved higher levels of competition performance (Baseball Professional Leagues—MLB) than their relatively older peers, according to the total number of drafted players 2. No influence of the RAE on long-term individual performance was observed, once the player reached high levels of competition (Major League Baseball—MLB)	Influence of RAE reversal on long-term individual performance
Steingröver et al. (2016)	To replicate previous findings on RAEs among NHL ice hockey players, NBA basketball players, and NFL football players and in a second step to investigate the influence of RA on career length in all three sports	Individual statistics throughout the sports career: no. of games	1. Relatively young players played more games throughout their professional NHL career. However, in the NBA and in the NFL, there was no such performance phenomenon 2. Considering the individual ranking, the relatively young NHL players with a medium/high individual ranking (positions 25–125th of 201) and relatively young NBA players with a medium/high individual ranking (positions 25–125th of 141), played more games than the relatively older players. No relationship was appreciated in the NFL players.	Influence of RAE reversal on long-term individual performance (NHL and NBA)No relationship between RAE and long-term individual performance (NFL)
Torres-Unda et al. (2016)	To compare anthropometric, maturational, and physical performance variables regarding the performance of the teams in a championship. In addition, another objective was to explore the relationship between maturity-related parameters, anthropometric variables, and physical performance variables of boys enrolled in elite basketball teams and the relationship between these parameters and their performance in basketball	Individual statistics:points per minute; points per game; index performance rating (PIR) and time played per game (min)Collective statistics:final team position	1. A relationship between chronological age/RA, when the player reached the maximum peak height velocity (YAPHV), and competition performance was observed, in terms of points scored and performance index rating (PIR). This relationship decreased when the results were weighted by the minutes played 2. The combination between an early maturation (years from age at peak height velocity) and advanced maturity status (relatively older age) was identified as a key factor to reach the highest levels of competition performance in basketball. Thus, relatively older players performed better than relatively young peers 3. Relatively older players were overrepresented in those basketball teams that performed better in competition based on the final position	Influence of RAE on short-term individual and collective performance
Fumarco et al. (2017)	First, we test for the presence of the RAE on points and on salaries with quantile regressions, which allow us to explore how the RAE varies along the distribution of points scored and salary. Second, we investigate the RAE on the quarter of birth distribution by draft age (i.e., 17, 18, 19), which is established by NHL drafting rules; this is the first time such analysis is conducted	Individual statistics:points (goals + assists)	1. The relatively young drafted players scored more points (goals + assists) in competition than their relatively older peers. The difference increased when it was considering those players with the best score ranking (>90%).	Influence of RAE reversal on long-term individual performance
Rubajczyk et al. (2017)	To identify the RAE in youth basketball games in Poland while taking into consideration the age, sex, and the players' match statistics. Additionally, the aim of this study is to determine whether differences in the body height of players are associated with the success of the team	Individual statistics:points per game; assists per game; rebounds per game; steals per game; blocks per game; turnovers per game; PIRCollective statistics:final team position	1. Relatively older players achieved higher individual performance parameters than relatively young players in U-14 men category. No impact of the RAE on competition performance was observed in the remaining male categories (U-16, U-18, and U-20) and in women 2. Relatively older players (with higher height) scored more points per game than relatively young players in male and female U-14 category 3. The teams with the worst classification in the men's competitions showed roster made up mainly of players with a bigger height differential between the relatively older players (Q1) and the relatively young peers (Q4) than the teams that performed better (final position)	Influence of RAE on short-term individual performance(male U-14)No relationship RAE and short-term individual performance (male U-16, U-18, and U-20 and female)Influence of RAE on short-term collective performance
Bjørndal et al. (2018a)	(a) To evaluate the prevalence of the RAE in international youth, junior, and senior Norwegian male and female handball players; and (b) explore the relationship between RA and the number of international youth, junior, and senior level appearances	Individual statistics:number of international appearances	1. The relatively older female players in the U-18 category were called up more times by the Norwegian national team than the relatively young peers. No impact of the RAE on the remaining female categories (U-20 and senior) or on male categories (U-19, U-21, and senior) was observed 2. Considering the long-term performance (number of international appearances) no impact of the RAE was found in those players who had already been previously selected at least once	Influence of RAE on long-term individual performance (female U-18)No relationship RAE and performance (male and female U-20 and senior)
Ibañez et al. (2018)	(i) To examine the distribution of birth dates in competitive basketball in the U-18 category, differentiating by playing position and (ii) to analyze the effect of the RAE on performance according to playing position using performance indicators	Individual statistics:points scored, tried and successful two- and three- point shots, tried and successful free throws, total rebounds, defensive and offensive rebounds, assists, steals, turnovers, blocks committed and received, dunks, personal fouls committed and received, PIR, and minutes played	1. Relatively older players, who occupied the “guard” position obtained higher competition performance in points scored, % effectiveness in 2-point shots and value of the PIR than their relatively young peers 2. Relatively older players, who occupied the “guard-forward” position performed better on blocks made than their relatively young peers 3. Relatively older players who occupied the “center” position reached higher competition performance in points scored, 2-point shots, and value of the PIR than their relatively young peers	Influence of RAE on short-term individual performance
Jones et al. (2018)	First, to test whether RAEs highlighted thus far extend beyond youth sport and elite sport into the world's “super elite” performers, whilst controlling for a significant limitation of previous research by considering intra sport differences through assessing RAE prevalence across the different positions. Second, to determine whether comparing RAE across different sports at the super-elite level will allow exploration of intersport differences	Study 1 Individual statistics:ranking among the 30-20-10 best players in the world, maintenance period (1 month to 5 years) in those positions for the last 10–20 yearsCollective statistics:international team ranking	Study 1: 1. Relatively older cricketers showed better individual performance indicators than their relatively young peers 2. Regarding the analysis by position, the relatively older cricketers, who occupied the “batsmen,” “spin bowler,” and “bowler combined” positions, were able to performance better (no. ranking and maintenance period in the ranking) than their relatively young peers. There was no impact of the RAE on performance in the players who occupied the “pace bowler” position	Study 1: Influence of RAE on long-term individual and collective performance
		Study 2 Individual statistics:number of international appearances; player career longevity and victory rateCollective statistics:international team ranking	Study 2: 1. Relatively young rugby players showed better competition performance than their relatively older peers 2. Regarding the analysis by position, relatively young rugby players, who occupied the “forward” position performed better than their relatively older peers. The impact was not found in the players who occupied the “back” position	Study 2: Influence of RAE reversal on long-term individual and collective performance
Yagüe et al. (2018)	To verify the RAE in the professional male soccer of the ten best national leagues of the UEFA Confederation during the 2016/2017 season, as well as to verify the possible differences and correlations between the RAE and the players' position, and the final classification	Collective statistics:final team position	1. The teams with the best (1st−4th position) and worst (last four positions) qualifying results showed a roster team with an overrepresentation of relatively older players. In the teams in the middle of the classification, the impact of the RAE on performance was weaker. The exception to this fact occurred in the Belgian Football League (Eerste Klasse) where no impact of the RAE on competition performance was identified	Influence of RAE on short-term collective performance(except Eerste Klasse)
Barrenetxea-Garcia et al. (2019)	(i) There would be an overrepresentation of players born in the first months of the year in elite water polo, (ii) there would be a larger percentage of left-handed players on the wing positions than in the general population, and (iii) RAE would be present in right-handed and not in left-handed water polo players	Individual statistics:played minutes, number of shots, number of goals, number of shots per minute, number of goals per minute, shots endured per minute, and blocks per minute	1. The RAE did not have a significant impact on any statistical performance parameter in water-polo players, both in men and women	No relationship between RAE and short-term individual performance
Lago-Fuentes et al. (2019)	To verify the occurrence and effect size of the RA in professional futsal players of the Spanish First Division, by observing how its presence and impact changed according to the season, the team level, and the player position	Collective statistics:final team position	1. The teams with the best qualifying results (play-off) or intermediate results (outside the play-off zone and the relegation zone) were made up of an overrepresentation of relatively young players. No impact of the RAE was observed in the teams at the bottom of the classification (promotion or relegation zone) 2. In the “goalkeeper” and “pivot” positions, an overrepresentation of relatively young players was confirmed	Influence of RAE on short-term collective performance(better classification)No relationship between RAE and short-term individual performance (worst classification)

*PIR (performance index rating) = a statistical formula also used by the FIBA, the Euroleague and the Eurocup, and various European national domestic leagues to determine the performance of each player in match (Arrieta et al., 2016)*.

#### Summary

**Figure 4:**
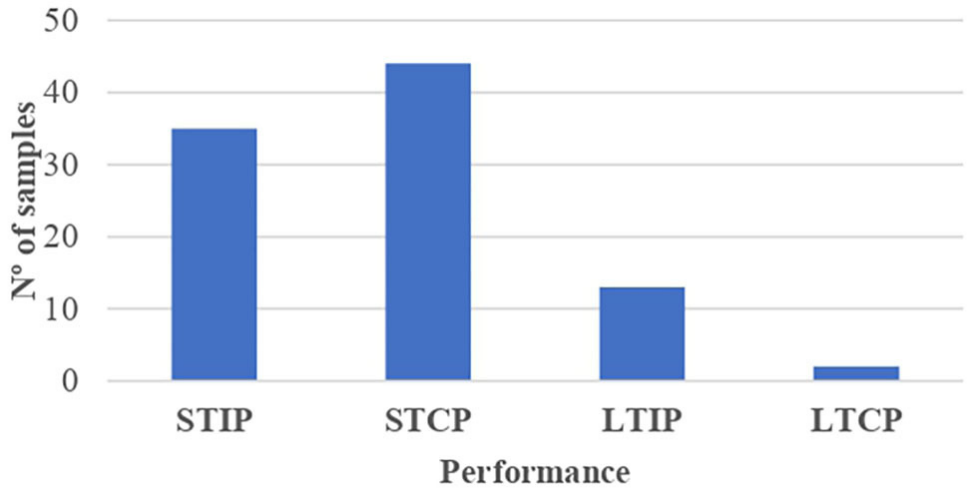
Performance indicators by samples based on the result in competition and the performance production period. STIP, short-term individual performance; STCP, short-term collective performance; LTIP, long-term individual performance; LTCP, long-term collective performance.

**Figure 5:**
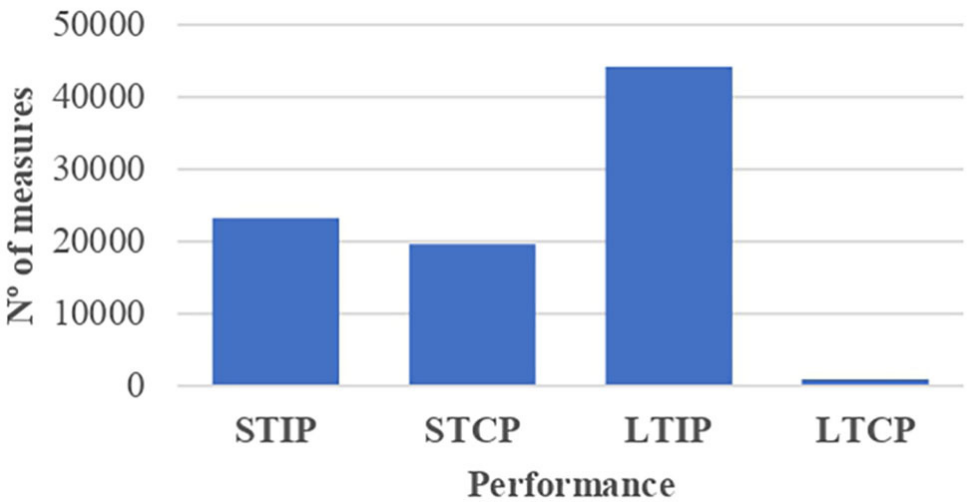
Performance indicators by measurements based on the result in competition and the performance production period. STIP, short-term individual performance; STCP, short-term collective performance; LTIP, long-term individual performance; LTCP, long-term collective performance.

Looking at the competition performance and taking as a reference the number of performance measurements made (Figures 4, 5), individual performance indicators were utilized in 76.61% of the measurements, whereas the collective performance indicators entailed the 23.39% of the total. Among the samples analyzed using individual performance indicators (*n* = 48), an unequal distribution of athletes was observed according to the performance production period. Thus, 23,240 performance measurements were identified based on the short-term results achieved through the consideration of official statistical parameters (26.41%), whereas 44,180 performance measurements based on the attainments reached throughout the sports career were registered (50.20%). With regard to the samples that analyzed the collective performance indicators (*n* = 46), 19,634 short-term measurements associated with the final team classification/position in competition were found (22.31%), whereas only 952 long-term measurements linked to international rankings and maintenance periods (1.08%) were observed.

Table 6 shows the relationship between RAE and competition performance based on the measurement indicators used (*n* = 87,556), in terms of the result (individual and collective) and the performance production period (short term and long term). The following findings were identified: (a) the correlation between competition performance and the effect of the athlete's birthdate occurred with greater force in those cases where RAE reversal was detected (52.64% measurements), whereas it was not so decisive in cases with presence of the RAE (30.36%). No relationship between RAE and competition performance was found (17%); (b) this connection, RAE and performance, was observed, to a greater degree, in the short-term collective performance measurements [30 samples (15,841 measurements)]; (c) regarding the relationship between RAE reversal and competition performance, the birthdate had a noticeable influence on individual performance over large periods of time [6 samples (43,523 measurements)]; (d) RAE had no clear impact on immediate or short-term individual performance (12 samples, 8,392 measurements); (e) measurements that yielded results on RAE reversal and the short-term individual performance were not found; (f) the impact of the RAE and RAE reversal was greater on long-term performance (52.57% of measurements) than on short-term performance (30.43% of measurements).

**Table 6:** Summary of samples (*n*) and performance measures (PM) [*n* and (%)] according to the relationship between relative age effect (RAE or RAE reversal) and competition performance (influence or no influence).

**Performance**		**Influence—RAE**		**Influence—RAE reversal**		**No influence**	
		**Samples**	**PM**	**Samples**	**PM**	**Samples**	**PM**
		***n***	***n (%)***	***n***	***n (%)***	***n***	***n (%)***
Performance (St)	IPI	11	8,935 (10.20)	0	0 (0)	12	8,392 (9.58)
	CPI	30	15,841 (18.09)	9	1,873 (2.14)	13	2,936 (3.35)
Performance (Lt)	IPI	3	1,546 (1.77)	6	43,523 (49.71)	7	3,558 (4.07)
	CPI	1	262 (0.30)	1	690 (0.79)	0	0 (0)

*n, absolute frequency; %, relative frequency; PM, performance measures; St, short-term; Lt, long-term; IPI, individual performance indicators; CPI = collective performance indicators*.

Further evaluating this relationship, based on constraints-based theoretical model, the most outstanding findings are shown in Table 7. In relation to sample characteristics (individual constraints): (1) according to “gender,” the short-term individual performance in male sports was the most affected by the RAE reversal (49.71% measurements), whereas the RAE had no impact on the competition performance in female sports, with regard to 16 samples (7.96% measurements); (2) considering the “age group,” the greatest influence of the RAE on competition performance was observed in the adult development stage, with 47,281 measurements (54.00%), whereas in adolescence only 5.76% of measurements were identified. The higher impact of the RAE (RAE reversal) on performance was found at the long-term individual level in adulthood [31,436 measurements (35.90%)]. Furthermore, this impact amplified as the chronological age of the athletes increased (adolescence, 0%; postadolescence, 4.46%; adult, 38.83%). On the other hand, the influence of the RAE was identified, mainly, on the short-term collective performance measurements (adolescence, 2.88%; postadolescence, 8.71%; adult, 6.50%).

**Table 7:** Summary of samples (*n*) and performance measures (PM) [*n* (%)] within the relationship between RAE and competition performance by characteristics of athletes (gender and age group) and sport context (type of sport, competition category and competition level).

**Performance**		**Influence—RAE**		**Influence—RAE reversal**		**No influence**	
		**Samples**	**PM**	**Samples**	**PM**	**Samples**	**PM**
		***n***	***n (%)***	***n***	***n (%)***	***n***	***n (%)***
**GENDER**							
Men							
Performance (St)	IPI	11	8,935 (10.20)	0	0 (0)	4	3,248 (3.71)
	CPI	26	12,422 (14.19)	9	1,873 (2.14)	7	1,352 (1.54)
Performance (Lt)	IPI	2	1,290 (1.47)	6	43,523 (49.71)	5	3,323 (3.80)
	CPI	1	262 (0.30)	1	690 (0.79)	0	0 (0)
Women							
Performance (St)	IPI	0	0 (0)	0	0 (0)	8	5,144 (5.88)
	CPI	4	3,419 (3.90)	0	0 (0)	6	1,584 (1.81)
Performance (Lt)	IPI	1	256 (0.29)	0	0 (0)	2	235 (0.27)
	CPI	0	0 (0)	0	0 (0)	0	0 (0)
AGE GROUP^*^							
Adolescence (12–14 years)							
Performance (St)	IPI	2	1,295 (1.68)	0	0 (0)	1	1,228 (1.59)
	CPI	3	2,523 (3.27)	0	0 (0)	0	0 (0)
Performance (Lt)	IPI	0	0 (0)	0	0 (0)	0	0 (0)
	CPI	0	0 (0)	0	0 (0)	0	0 (0)
Postadolescence (14–19 years)							
Performance (St)	IPI	4	1,490 (1.93)	0	0 (0)	8	5,550 (7.19)
	CPI	15	7,630 (9.88)	0	0 (0)	7	1,774 (2.30)
Performance (Lt)	IPI	2	1,284 (1.66)	2	3,901 (5.05)	4	3,276 (4.24)
	CPI	0	0 (0)	0	0 (0)	0	0 (0)
Adult (>19 years)							
Performance (St)	IPI	3	4,012 (5.19)	0	0 (0)	3	1,614 (2.09)
	CPI	12	5,688 (7.36)	9	1,873 (2.43)	6	1,162 (1.50)
Performance (Lt)	IPI	1	262 (0.34)	3	31,436 (40.70)	3	282 (0.37)
	CPI	1	262 (0.34)	1	690 (0.89)	0	0 (0)
**TYPE OF SPORT**							
Invasion games							
Performance (St)	IPI	11	8,935 (10.20)	0	0 (0)	12	8,392 (9.58)
	CPI	30	15,841 (18.09)	9	1,873 (2.14)	13	2,936 (3.36)
Performance (Lt)	IPI	2	1,284 (1.47)	5	13,323 (15.22)	7	3,558 (4.06)
	CPI	1	262 (0.30)	1	690 (0.79)	0	0 (0)
Striking and fielding games							
Performance (St)	IPI	0	0 (0)	0	0 (0)	0	0 (0)
	CPI	0	0 (0)	0	0 (0)	0	0 (0)
Performance (Lt)	IPI	1	262 (0.30)	1	30,200 (34.49)	0	0 (0)
	CPI	0	0 (0)	0	0 (0)	0	0 (0)
**COMPETITION CATEGORY**							
U-14 to U-18 (youth categories)							
Performance (St)	IPI	6	2,785 (3.18)	0	0 (0)	7	5,687 (6.50)
	CPI	17	9,361 (10.69)	0	0 (0)	6	1,475 (1.68)
Performance (Lt)	IPI	1	256 (0.29)	0	0 (0)	1	299 (0.34)
	CPI	0	0 (0)	0	0 (0)	0	0 (0)
U-20 to U-22 (junior categories)							
Performance (St)	IPI	1	384 (0.44)	0	0 (0)	3	1,460 (1.67)
	CPI	4	1,729 (1.97)	0	0 (0)	3	581 (0.66)
Performance (Lt)	IPI	0	0 (0)	1	8,186 (9.35)	2	372 (0.42)
	CPI	0	0 (0)	0	0 (0)	0	0 (0)
Senior category							
Performance (St)	IPI	4	5,766 (6.59)	0	0 (0)	2	1,245 (1.42)
	CPI	9	4,751 (5.43)	9	1,873 (2.14)	4	880 (1.01)
Performance (Lt)	IPI	2	1,290 (1.47)	5	35,337 (40.36)	4	2,887 (3.30)
	CPI	1	262 (0.30)	1	690 (0.79)	0	0 (0)
**COMPETITION LEVEL**							
National							
Performance (St)	IPI	6	7,061 (8.06)	0	0 (0)	7	6,045 (6.90)
	CPI	18	12,091 (13.82)	9	1,873 (2.14)	0	0 (0)
Performance (Lt)	IPI	1	1,028 (1.17)	5	42,833 (48.92)	2	2,787 (3.18)
	CPI	0	0 (0)	0	0 (0)	0	0 (0)
International							
Performance (St)	IPI	5	1,874 (2.14)	0	0 (0)	5	2,347 (2.68)
	CPI	12	3,750 (4.28)	0	0 (0)	13	2,936 (3.35)
Performance (Lt)	IPI	2	518 (0.60)	1	690 (0.79)	5	771 (0.88)
	CPI	1	262 (0.30)	1	690 (0.79)	0	0 (0)

n, absolute frequency; %, relative frequency; PM, performance measure; St, short term; Lt, long term; IPI, individual performance indicators; CPI, collective performance indicators.

* *Because of the lack of data about the age of participants in some studies, the analysis of the sample by age group was reduced to 77,232 athletes*.

Examining the sport context (task constraints), the findings were the following: (1) with regard to the “type of sport,” the RAE showed a longer influence on long-term collective performance in the “invasion games” [15,841 measurements (18.09%)], whereas the RAE reversal had a higher impact on long-term individual performance [13,323 measurements (15.22%)]. However, a considerable number of samples (n = 32) with no relationship between the RAE and competition performance were identified. In this set of samples, 8,392 short-term individual performance measurements were registered (9.58%). In the “striking and fielding games,” according to 30,200 measurements (34.49%), the performance most affected by the RAE reversal was the long-term individual performance; (2) with regard to the “competition category,” a transition process from the youngest categories, where a greater influence of the RAE on competition performance was identified (12,402 measurements; 14.16%), to the higher categories, in which a bigger impact of the RAE reversal on the long-term competition performance was detected [junior: 8,183 measurements (9.35%); senior: 37,900 measurements (43.29 %)]; (3) from the perspective of the “competition level,” at the national level, the long-term competition performance was more affected by the RAE reversal [43,861 measurements (50.09%)], whereas the results found were mixed at the international level, showing no prevailing relationship between RAE and competition performance. However, a noticeable number of samples (n = 23) revealed no relationship between the RAE and competition performance at the international level (6,054 measurements; 6.91%).

## Discussion

**Figure 6:**
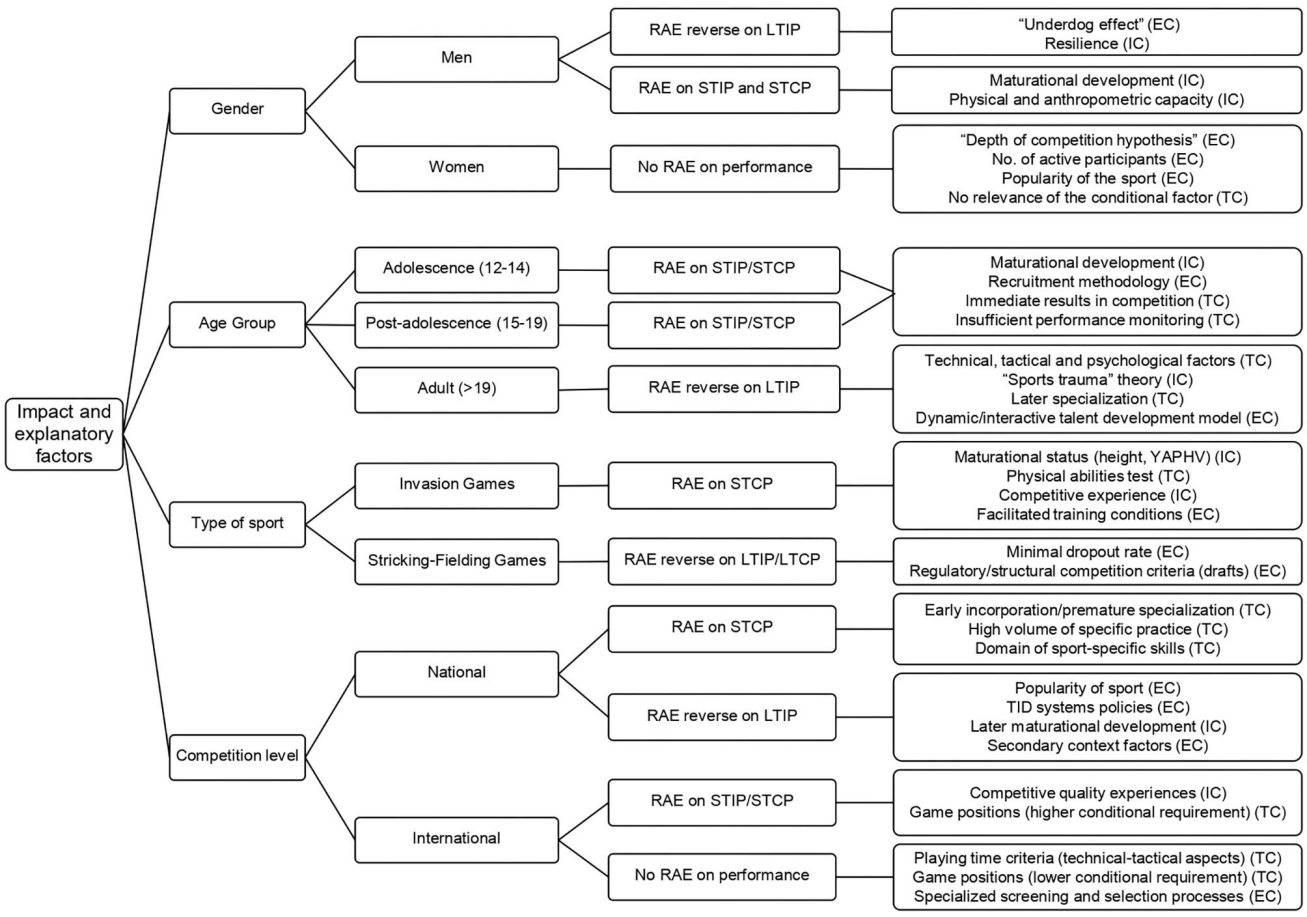
Summary of impact and explanatory factors of the influence/non-influence of the RAE/RAE reversal on the competition performance in team sports by categories and sub-categories according to Wattle's constraints-based theorical model. STIP, short-term individual performance; STCP, short-term collective performance; LTIP, large-term individual performance; LTIC, large-term collective performance; IC, individual constraints; TC, task constraints; EC, environmental constraints.

The present study represents the first attempt to synthesize and analyze the scientific evidence regarding the impact of the RAE and its relationship with competition performance in team sports. Based on the analyzed data, the results confirmed (i) a prominent influence of the RAE on competition performance in team sports in 83% of the measurements and (ii) a greater impact of the RAE/RAE reversal on short-term collective performance and on long-term individual performance (sport career). Moreover, attending to the constraints-based theoretical model, individual constraints, sample characteristics (gender and age group), and task constraints, sport context (type of sport, competition level, and competition category), were modifying factors of the impact of the RAE and its influence on competition performance (Figure 6).

However, it is necessary to minimize the results yielded because of the difficulty of establishing a homogeneous discussion and common conclusions, given the high degree of variability that the investigations showed in their study design. Therefore, for a correct interpretation of the existing scientific literature in this regard, characterized by a lack of homogeneity in the methodological field (very diverse study designs applied to very different samples, in terms of age/age group and competitive category), it is necessary to contextualize the investigations without advancing hasty conclusions.

### RAE and Competition Performance (Study Quality Assessment)

With regard to the study quality analysis, it was identified that the investigations that yielded better quality scores, according to the adapted version of the STROBE checklist (Vandenbroucke et al., 2014; Smith et al., 2018), were associated with the analysis and evaluation, mainly, of individual short-term (statistical parameters) and long-term (attainments throughout the sport career) performance indicators. However, it could not be confirmed that a high study quality score was linked to a specific trend in terms of the impact of the RAE on competition performance, producing results with great variability and heterogeneity. These findings highlight the need to provide complete data on the characteristics of the participants and the context for a complete and in-depth analysis. The study quality evaluation list can be a valid and useful tool for subsequent works that aim to establish a connection between the relative age and competition performance.

### RAE and Competition Performance by Gender

According to the gender of the athletes analyzed (individual constraints), in men, a higher impact of the RAE reversal on competition performance was observed, especially on individual long-term performance (sport career), whereas in the case of women, the presence of the RAE was detected in some samples; however it did not have, for the most part, an impact on performance.

These results, in men's sport, are in line with other studies that confirmed that relatively young players, considered as “talented,” achieved more and greater attainments throughout their sport career in terms of competitive experience (Carling et al., 2009), competitive productivity (Sims and Addona, 2016), longevity of sport career (Jones et al., 2018), ranking position (Ford and Williams, 2011), or salary (Ashworth and Heyndels, 2007). These results, given the relevance of team sports in their respective sociocultural contexts, could be explained by the “underdog effect” (Gibbs et al., 2012). The fact of being born in the last months of the year would allow the development and acquisition of specific technical–tactical skills, which would help the relatively young players overcome physical and anthropometric limitations. Moreover, greater experimentation of stressful training situations under pressure in youth categories (Andronikos et al., 2016), even with some need to face potentially positive adverse experiences— “traumas” — (Collins and MacNamara, 2017), along with a great effort in the learning process (Roberts and Stott, 2015), could suppose that relatively young athletes overcome, to a greater extent, the challenges presented throughout their sport career, displaying greater resilience than relatively older players (McCarthy and Collins, 2014; McCarthy et al., 2016).

On the contrary, a strong impact of the RAE on the selection process and individual and collective competition performance was identified (Vaeyens et al., 2005a,b; Yagüe et al., 2018). This reality can be explained by the performance production period. The analysis of these studies was based on short-term performance measurements (statistical parameters in competition), determining “competitive” as meaning having a roster composed of a majority of relatively older players due to a greater maturational development, which was reflected in higher anthropometric and physical patterns (Gastin and Bennett, 2014).

In women's sport, it seems that, even with an overrepresentation of relatively older players, the relative age did not entail an influence on competition performance. Probably, as the magnitude of the RAE in female sport is less than in male sport (Smith et al., 2018; de la Rubia et al., 2020), because of factors such as the depth of the competition (Baker et al., 2009) or the number of active participants and the popularity of the sport (Sedano et al., 2015), this phenomenon is not relevant enough to affect the competition performance, either individually or collectively. Furthermore, because of the conditional component of the players seeming to be less decisive for achieving high performance in team sports (Konstantinos et al., 2018), the biological differences (physical, anthropometric, physiological, etc.) that could be derived from the RAE would be reduced, and therefore, relative age would not be a relevant factor in women's performance.

### RAE and Competition Performance Throughout the Age Group—Competition Category

Considering jointly the age group and competition category (task constraints), even though the cases in which there was an influence of the RAE on competition performance were very frequent, it was detected that the impact of the RAE on competition performance decreased gradually as the chronological age of the players increased, that is, when the sport transition by age categories is taking place. Therefore, an impact of the RAE on short-term performance indicators was observed in adolescent and post-adolescent athletes (youth and junior categories), whereas in the adulthood or senior category the long-term individual competition performance was affected by the RAE reversal.

The gradual reduction of the impact of the RAE and therefore its lower weight on the performance of athletes in the higher competition levels (senior categories) seem to be explained from two perspectives: (1) by the maturational development of the athletes, that is, the physical and anthropometric advantages that relatively older players have in the early stages of sport development (adolescence) would tend to equalize in adulthood in relation to the relatively young peers (Leite et al., 2013); (2) because of the complexity of considering and measuring the performance in team sports. The possible maturational advantages would not be so decisive in advanced stages of development and superior performance categories. Thus, the lower relevance that conditional capacities exert on the competition performance, to the detriment of the technical, tactical, strategic, and even psychological qualities (Rampinini et al., 2007), could suppose a reduced impact of the RAE; and (3) because of the trauma connected to talent (Collins and MacNamara, 2012). The difficulties, derived from the RAE, that relatively young athletes would have to overcome in their early stages of development (i.e., expectations breach, the non-selection for a team, or change of training group), could cause them to develop determining psychological abilities to achieve high performance in adult age or senior stages (Collins et al., 2016; Savage et al., 2017). Even some studies (Collins and MacNamara, 2012; Sarkar and Fletcher, 2014) demonstrated the “need” for these athletes to experience trauma to reach professional sports levels. Therefore, it seems that to be born in the last months of the year would not suppose a disadvantage to progress toward high sport performance.

According to the performance production period, a higher short-term performance by relatively older players in early development stages could correspond to various reasons, apart from the maturational process. With regard to the recruitment methodology, it seems that coaches prioritize the consideration of immediate performance indicators in talent identification programs for elite levels, trying to predict, in this way, the athlete's sport development (Simonton, 2001). Therefore, the selection processes seem to be biased in favor of the relatively older players. Nevertheless, it is necessary to highlight that a large part of the scientific literature in this regard provides us with studies carried out in top-level international contexts (Rubajczyk et al., 2017; Carraco et al., 2020), so it seems logical to think that the impact of the RAE would have an exponential influence on short-term competition performance. Thus, the most employed criteria in talent identification and development (TID) systems, in stages prior to the highest competitive level (adulthood–senior category), are short-term indicators, neglecting other important ones in team sports, such as the specific criteria of the game (decision-making, leadership, cognitive skills, etc.) (Hyllegard et al., 2001).

Furthermore, insufficient and deficient monitoring of performance indicators could cause an imbalance in the athlete's sports development without considering the individual's characteristics (Hartwig et al., 2009). However, in adulthood, corresponding to the senior category, the relatively young players yielded better results in the long-term performance indicators (Gil et al., 2019). A late specialization, as happens in most team sports, and a dynamic and interactive model of sports talent development based on comprehensive learning activities seem to be factors that modulate long-term performance, favoring longevity and quality of the sport career (Güllich and Emrich, 2014). Moreover, although scientific evidence is limited, it was suggested that specialized environments based on talent detection and selection processes, in which the maturational development of the athlete is ignored, could be correlated with shorter and less successful sports careers.

### RAE and Competition Performance by Type of Sport

In the analysis carried out regarding the type of sport (task constraints), the performance results in the “invasion games” were influenced by the presence of the RAE, whereas the impact of the RAE reversal was observed in the “striking and fielding games.” Within the first group, in sports such as football/soccer (Williams, 2010; González-Víllora et al., 2015) and basketball (Arrieta et al., 2016; Rubajczyk et al., 2017), a clear relationship between the RAE and short-term collective performance was confirmed. The scientific literature coincides in highlighting the difference in the maturational status of athletes as a decisive factor. In that regard, Torres-Unda et al. (2016) found higher values in maturation indicators, such as height or the “years at peak of high velocity,” in relatively older players than their relatively younger peers. Furthermore, relatively older players produced better results in tests associated with physical capacity, which translated into better competition performance and therefore a better final team classification (Augste and Lames, 2011). However, if the performance is discriminated as positive or negative, the relationship between RAE and competition performance does not seem to behave in the same way. In this context, Yagüe et al. (2018) verified in their study on the 10 best European football leagues that teams in the middle or lower part of the classification composed of a high percentage of relatively older players achieved a better final position. Conversely, the relationship between RAE and short-term collective performance in the top teams disappeared.

Furthermore, in “invasion games,” the increased competitive experience of relatively older players seems to be another key point. Thanks to an early identification of talent in athletes born in the first months of the year due, partially, to a greater probability of selection (Helsen et al., 1998), relatively older players would tend to enjoy better training conditions (sport facilities, coaches, etc.) (Hancock et al., 2013). This would help to increase their competitive experience, both qualitatively and quantitatively, which would translate into greater individual and therefore collective performance in team sports (Williams, 2010).

On the other hand, in the “striking and fielding games” (baseball and cricket), an RAE reversal was detected. The scientific literature in this regard is not plentiful, especially in cricket, leading to a lack of depth in the analysis of this phenomenon. This could affect the impact of the results. Unlike the “invasion sports,” baseball and cricket, among others, are disciplines where physical and physiological maturation and subjective performance evaluation are not considered as relevant (Zuma et al., 2017). This fact would cause the dropout rate of relatively young athletes to be low, and therefore, they could develop their potential without biased selection processes based on their birthdate (Jones et al., 2018).

Moreover, in sports such as baseball, the normative criteria of the structure of the competition (drafts) seem to favor the presence of RAE reversal. In these athlete allocation processes, the relatively young players, because of selection based on short-term and biased performance identification criteria, are not usually chosen in the first instance by the top teams. This fact would suppose that they continued playing at a lower competitive level, enjoying more “quality” time played (Thompson et al., 1992). Therefore, a late pick in the draft of these kinds of sports, in the case of relatively young players, could mean a higher long-term competition performance (Sims and Addona, 2016), reaching maximum individual levels of success, such as most valuable player (Ford and Williams, 2011).

### RAE and Competition Performance by Competition Level

In the analysis regarding the competition level (task constraints), an influence of the RAE on performance in national competitions was found, whereas a lack of impact of the RAE or RAE reversal on competition performance in international contexts was observed. In the domestic sphere, in terms of the number of measurements carried out, the RAE showed a considerable influence on short-term collective performance (final team position). Although there is no clear evidence of this relationship in the scientific literature, the reasons responsible for this considerable influence could be found in the athlete's development toward high performance. With a short-term performance objective in formative categories, it seems that an early incorporation and a quick specialization in the sport, a high volume of specific practice, and a high domain of specific skills lead to the achievement of a strong long-term individual and collective performance (Weissensteiner et al., 2008). According to this research line, Augste and Lames (2011) detected that those teams from the three best U-17 development leagues in Germany, mainly composed of relatively older players, achieved a final classification 1,035 positions better than the other teams. Similar results were found in the U-17 teams of the German Bundesliga clubs in the 2010–2011 and 2011–2012 seasons (Grossmann and Lames, 2013).

In addition, the long-term individual performance at the national level was affected by the RAE reversal. The study samples, in which this phenomenon was found, were observed for the most part in professional team sports in the United States, such as baseball or ice hockey (Deaner et al., 2013; Sims and Addona, 2016; Steingröver et al., 2016; Fumarco et al., 2017). The great popularity of these sports specialties in a particular geographic context, different policies connected to TID systems, the decrease in maturational differences, and secondary factors (family) throughout the university stage seem to become key environmental factors in justifying that relatively young athletes usually enjoy more successful sports careers than their relatively older peers (Wattie et al., 2015). Furthermore, Ashworth and Heyndels (2007) demonstrated in a study with top-level German football players that relatively young athletes earned systematically higher wages than their relatively older peers.

The results regarding the relationship between RAE and performance in international competitions were mixed. Although most of the performance indicators focused on the short term due to the duration of the competition, normally reduced, a similar number of measurements was registered between samples that showed an impact of the RAE on competition performance and samples that presented a lack of connection between these variables. However, no strong evidence confirming the influence of the RAE on long-term competition performance was found. On the one hand, it seems that relatively older athletes, having lived more competitive quality experiences, could reach higher performance (Bjørndal et al., 2016). In contrast, Karcher et al. (2014) checked that the criteria used by coaches to decide the playing time of their players were based on technical–tactical considerations rather than aspects derived from RAE. Taking both realities into account, it would be important to introduce a modulating factor into the relationship between RAE and performance: the playing position. In team sports, individual performance is not achieved in the same way, but will largely depend on the playing position. For this reason, many studies made this distinction (Schorer et al., 2009b; García et al., 2014; Ibañez et al., 2018; Yagüe et al., 2018; Lago-Fuentes et al., 2019; de la Rubia et al., 2020), concluding, generally, that relatively older players showed a better performance in those positions with greater physical and anthropometric requirements (i.e., pivots in basketball, backs in handball or midfielders in football), whereas in other playing positions, less conditioned by the biological and maturational development (i.e., shooting guard in basketball; wings in handball or forwards in football), there was no difference in competition performance depending on the relative age.

### Unexpected Finding

The most significant unexpected finding was found in the performance analysis by competition level. Although some samples showed an influence of the RAE on competition performance at international level (Williams, 2010; Torres-Unda et al., 2016; Ibañez et al., 2018), interestingly, a similar number of samples and athletes, in which the RAE or RAE reversal did not affect competition performance, was also found (García et al., 2014; Karcher et al., 2014; Barrenetxea-Garcia et al., 2019), especially in the short term. Nevertheless, it should be specified that most of the measurements were collected from competitions in the senior category.

It seems that the primary mechanism that transforms and promotes the RAE as a bias factor appears in the early stages of the athlete's development process (Cobley et al., 2008; Schorer et al., 2009a). So, in adulthood and senior categories, the RAE, although present, does not have as much impact on competition performance. Furthermore, the screening and selection processes at these competitive levels are more specialized, being made up of sport-specific criteria and therefore usually escaping the influence of the RAE (Schorer et al., 2009a). This could suppose, as Karcher et al. (2014) affirm, that all players had the same opportunities to play, with no differences regarding the number of minutes played. Therefore, the RAE, as occurs in some studies in this review (González-Víllora et al., 2015; Bjørndal et al., 2018a), is not considered as the key factor that can modify and modulate competition performance.

## Conclusions

Competitive performance in team sports is affected by the RAE. The results highlighted, mainly, the impact of the RAE on the individual and collective athlete's performance in the short term, that is, regarding statistics parameters (individual performance) and final team classification (collective performance), particularly in youth and junior categories, which correspond to adolescence and postadolescence. On the other hand, a correlation between a higher long-term competition performance, primarily at the individual level, and being a relatively young athlete, that is, RAE reversal, was verified.

With regard to the analysis of the sample characteristics, the RAE or RAE reversal affected mainly men's competition performance, whereas this relationship was not marked in women's sport, in which a lack of influence of RAE or RAE reversal on competition performance was identified. By age group, a tendency to decrease the magnitude of the impact of the RAE or RAE reversal on competition performance as the athlete's chronological age increased was detected.

Regarding the analysis of the sport context, a greater impact of the RAE on competition performance was observed in the “invasion games,” whereas in the “striking and fielding games,” a greater influence of the RAE reversal was identified. By “competition category,” effects similar to those detected in the subcategory of “age group” were found. In other words, the impact of the RAE on competition performance was transforming as the category was superior, appreciating an RAE, to a greater extent, in lower categories (from U-14 to U-18); and an RAE reversal predominantly in junior categories (from U-20 to U-22). A notable lack of influence of the RAE on the individual and collective performance in the short and long term was detected in youth categories, rather than in the junior and senior categories. By “competition level,” the RAE showed a clear influence on short-term performance, both individual and collective, at national levels, whereas the RAE reversal affected long-term individual competition performance. At international level, the results were mixed, with no clear conclusions being established in this regard.

According to this scientific evidence, these findings should be considered by sport institutions and organizations, within the design and implementation of TID programs, so as to reduce possible selection biases of athletes caused by the RAE or RAE reversal, which, subsequently, could impact on competitive performance. A reduction in the influence of the RAE on competition performance could mean greater equality of opportunity for the athletes regardless of their birthdate within the same year of selection. Thus, the sport development process of relatively young athletes would be optimized by aligning individual constraints (sample characteristics) and task constraints (sport context) and thereby adjusting the athlete's participation throughout the sport career, especially in formative stages or youth categories.

## Limitations

First, it is possible that some published scientific evidence, especially as of January 2020, has not been identified and registered in this review despite the exhaustive systematic search process carried out. Second, the lack of some relative data, especially with regard to the age of the athletes, has meant that some samples were categorized as “not encodable” by “age group” category. Third, the present study, although not its main objective, does not provide specific data on the effect size of the RAE. Fourth, in order to carry out an in-depth analysis and because of the different methodologies employed to evaluate competition performance, the total number of participants (n = 77,329), which constituted the 77 samples, did not match with the number of measurements made on the performance of these athletes (n = 87,556). Therefore, the results must be accurately interpreted. Fifth, the great sampling and methodological diversity of the analyzed studies has entailed an increase in the degree of difficulty in extracting the main results and conclusions connected to the purpose of the systematic review. Sixth, because of the existence of some actions of the game that cannot be accounted for, but that are considered of great value by the coaches (intangibles), it would be convenient to minimize the scientific evidence identified.

## Practical Applications

Given that the athlete's training process is very complex, dependent on a multitude of factors, non-linear and even random, talent detection, development, and selection models should observe with greater caution the relationship between RAE and performance in competition. Thus, these models should try not to evaluate this problem by means of a dichotomous decision, but as a process in which time helps to make better decisions with the main objective of ensuring that athletes always remain involved in their sport discipline and assuming that their level performance can vary over time, and therefore, their participation in different competitive levels could also do so.

According to the previously mentioned objective and findings of this systematic review, guidelines or suggestions are proposed for coaches, stakeholders, and practitioners on how to approach and carry out the identification and development talent process with the aim of reducing the bias produced by the RAE: (1) do not focus the talent detection and recruitment on short-term parameters that yield a biased vision of the future potential of athletes; (2) carry out long-term monitoring of performance indices in relation to the maturational level of the athlete, allowing an adjusted and parallel development of the sport performance and maturational status; (3) collect a comprehensive database of athletes in terms of constraints-based theoretical model (gender, age group, competition category, sport, and competition level), which allows predicting longitudinal trends in the RAE's behavior with the aim to reduce its impact with regard to the athletes' characteristics and/or sport context; (4) organize development leagues or short competitions in national sport contexts that include those relatively young players not selected so that their training and competitiveness are not greatly affected; (5) adjust the participation of relatively older athletes in youth categories so as not to cause an overload of practice that may lead to a higher injury rate. This is to ensure that the long-term performance of relatively older athletes, especially in international sport contexts, is not adversely affected; (6) include psychosocial factors in the selection tests to link and connect the training, leadership, and cognitive skills variables. Thus, a balanced development of athletes will be ensured by reducing the influence of the RAE, (7) adjustment of age categories used in youth and international junior competitions, so that the relatively older players in a group can change their category every year.

## Future Research

According to a more in-depth analysis, the subsequent studies should include a meta-analysis that would provide more accurate statistical information on the behavior of the RAE on competition performance, considering the heterogeneity and complexity that the different performance indicators present in their measurement. On the other hand, expanding this research to other sport contexts (elementary competitive levels, such as local, regional, etc.) could help to compile a complete map of the impact and influence of the RAE on competition performance and, in this way, be able to adjust sports talent identification policies in a greater number of team sports and competitive levels. Furthermore, an in-depth evaluation of athletes in relation to competition performance, not only taking into account their primary characteristics, but also focusing on factors that are considered as secondary (coaches, friends, family, etc.), could help to carry out a holistic evaluation of sport talent, understanding the athlete as a whole composed of unequal parts, but all of them important to achieving the high performance.

## Author Contributions

AR, AL, and JL-C conceptualization, formal analysis, methodology, project administration, supervision, validation, visualization, and writing—review and editing. AR: data curation, investigation, resources, software, and writing—original draft preparation. All authors contributed to the article and approved the submitted version.

## Conflict of Interest

The authors declare that the research was conducted in the absence of any commercial or financial relationships that could be construed as a potential conflict of interest.
